# Time-Dependent Decay of mRNA and Ribosomal RNA during Platelet Aging and Its Correlation with Translation Activity

**DOI:** 10.1371/journal.pone.0148064

**Published:** 2016-01-25

**Authors:** Catherine Angénieux, Blandine Maître, Anita Eckly, François Lanza, Christian Gachet, Henri de la Salle

**Affiliations:** 1 UMR_S949, INSERM, Strasbourg, France; 2 Etablissement Français du Sang-Alsace (EFS-Alsace), Strasbourg, France; 3 Fédération de Médecine Translationnelle de Strasbourg (FMTS), Strasbourg, France; 4 Université de Strasbourg, Strasbourg, France; Medical Faculty, Ludwig Maximilians University Munich, GERMANY

## Abstract

Previous investigations have indicated that RNAs are mostly present in the minor population of the youngest platelets, whereas translation in platelets could be biologically important. To attempt to solve this paradox, we studied changes in the RNA content of reticulated platelets, i.e., young cells brightly stained by thiazole orange (TO^bright^), a fluorescent probe for RNAs. We provoked in mice strong thrombocytopenia followed by dramatic thrombocytosis characterized by a short period with a vast majority of reticulated platelets. During thrombocytosis, the TO^bright^ platelet count rapidly reached a maximum, after which TO^dim^ platelets accumulated, suggesting that most of the former were converted into the latter within 12 h. Experiments on platelets, freshly isolated or incubated *ex vivo* at 37°C, indicated that their “RNA content”, here corresponding to the amounts of extracted RNA, and the percentage of TO^bright^ platelets were positively correlated. The “RNA Content” normalized to the number of platelets could be 20 to 40 fold higher when 80–90% of the cells were reticulated (20–40 fg/platelet), than when only 5–10% of control cells were TO^bright^ (less than 1fg/platelet). TO^bright^ platelets, incubated *ex vivo* at 37°C or transfused into mice, became TO^dim^ within 24 h. E*x vivo* at 37°C, platelets lost about half of their ribosomal and beta actin RNAs within 6 hours, and more than 98% of them after 24 hours. Accordingly, fluorescence *in situ* hybridization techniques confirmed the presence of beta actin mRNAs in most reticulated-enriched platelets, but detected them in only a minor subset of control platelets. *In vitro*, constitutive translation decreased considerably within less than 6 hours, questioning how protein synthesis in platelets, especially in non-reticulated ones, could have a biological function *in vivo*. Nevertheless, constitutive transient translation in young platelets under pathological conditions characterized by a dramatic increase in circulating reticulated platelets could deserve to be investigated.

## Introduction

The major primary hemostatic functions of blood platelets (PLTs) imply a series of rapid signal transduction events generally completed within a few seconds or minutes [1]. PLTs can also participate in vascular inflammation, through mechanisms which are less well characterized in terms of their kinetics, especially *in vivo* [2]. The idea that PLTs might possess a functional translation system is attractive since according to this view, these cells would play a role in longer term cellular and immunological responses [3]. Translation in PLTs has been documented for nearly half a century [4]. However, its reality has been questioned on the grounds of the possible presence of contaminating leukocytes in PLT preparations [5]. Moreover, human PLTs contain rather small amounts of RNA [6], which would question its biological function.

Staining of PLTs with new methylene blue, a rough endoplasmic reticulum (RER) dye, allows the identification of a minor RER-rich population known as reticulated PLTs (retPLTs) [7]. This population can also be distinguished by flow cytometry (FC) after staining with thiazole orange (TO), a fluorescent probe for double stranded RNA molecules [7]. Animal studies have shown that TO^bright^ PLTs are relatively young, being less than 36 h old [8,9], whereas the PLT life span is 5 or 10 days in mouse and human, respectively; so, they represent a minor population, between less than 10% and a few percent of all PLTs, respectively. Increased TO^bright^ PLT counts have been associated with acute blood loss or reactive thrombopoiesis, in particular in cases of idiopathic thrombocytopenic purpura [10,11]. In this latter pathological context, transmission electron microscopy (TEM) confirmed that retPLTs contained RER, while metabolic labeling indicated that protein synthesis occurred in these cells [12].

Up until now, the precise characterization of the RNA content of PLTs has been neglected because PLTs were predominantly considered to be vestigial remnants of megakaryocytes (MKs). The experimental evidence that translation can occur in PLTs nevertheless challenged this view and incites one to better characterize their RNA content. In this objective, we studied the time-dependent changes of the “RNA content” of PLTs (defined as the RNA molecules extracted from PLTs), the integrity of their rRNA, the life span of beta actin mRNA in platelets placed in *ex vivo* conditions and, the constitutive biosynthetic activity in PLTs. Steady state conditions were compared with experimental conditions characterized by the transient presence of a vast majority of retPLTs. Our results were then correlated with previously published genetic studies of PLTs to assess the biological relevance of the deduced relative amounts of specific mRNAs in PLTs. Our experimental system also provides a unique means of investigating *in vivo* the functional characteristics of retPLTs.

## Results

### Ablation of megakaryocytes (MKs) and experimental non-immune thrombocytopenia

**Fig 1:**
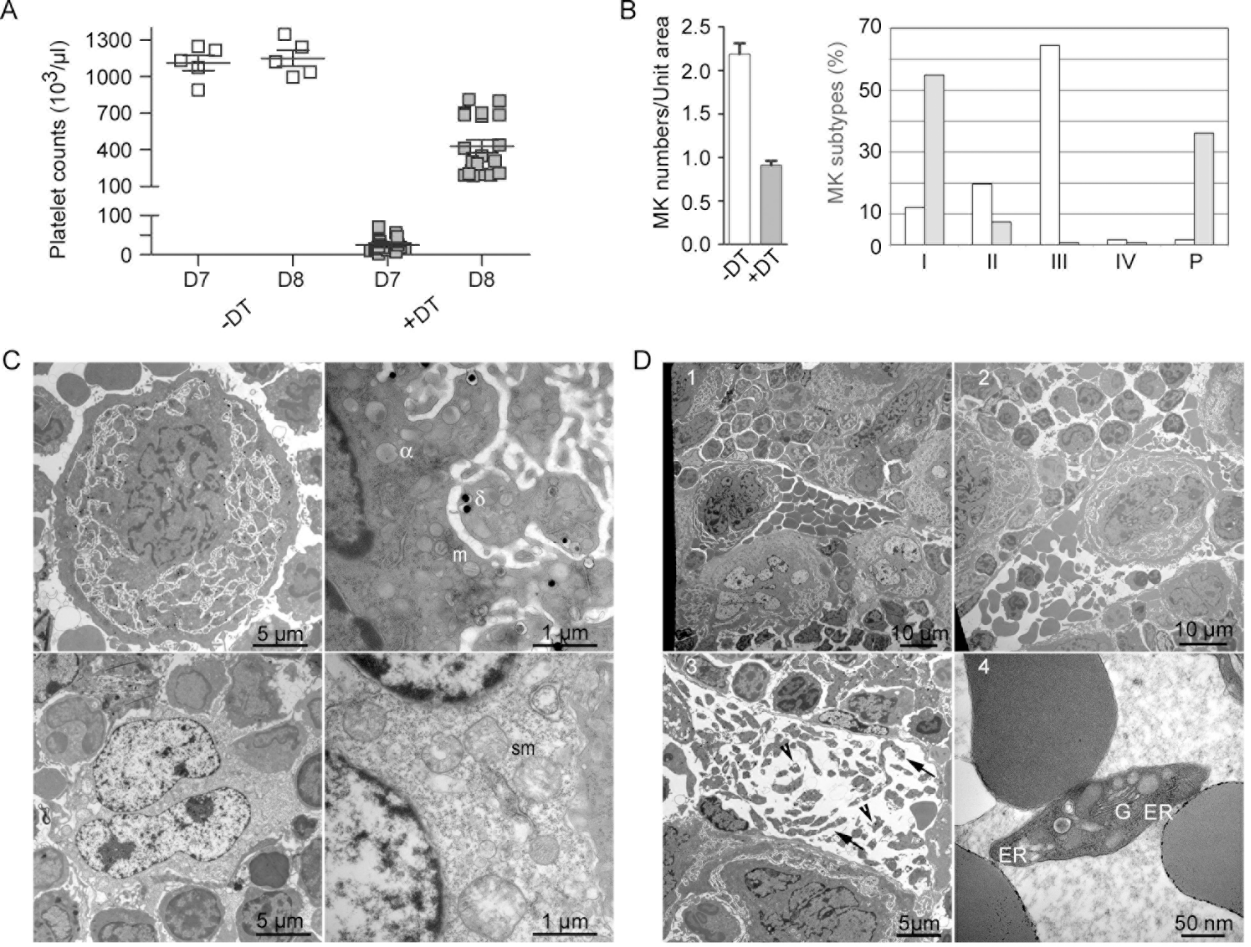
Enhanced megakaryopoiesis and thrombopoieisis after DT-mediated MK ablation. F1 iDTR x PF4-Cre mice received 100 ng/day DT or saline for 4 days (first injection on day 1). PLT counts decreased during the following days and PLT rebound occurred between days 7 and 8. (**A**) PLT counts of saline- and DT-treated mice (n = 5 and 20, respectively) were measured on day 7 and 8. Horizontal bars indicate the mean values of the counts. Comparing the different conditions, a two way ANOVA analysis followed by a Bonferroni post-test indicated a p value of <0.001, except for saline treated samples on days 7 and 8, which displayed no significant differences. (**B**) On day 5, bone marrow samples from 3 saline- and 3 DT-treated mice, with two sections per sample, i.e. 6 sections per condition, were analyzed by TEM; total numbers of 172 and 133 MKs were observed, respectively. MKs were classified according to their morphology, either as classical stage I to IV, or as pycnotic cells. Left, the density of MKs per unit area (14,400 μm2) was determined on sections from untreated and treated mice; the histograms represent the mean distribution and standard deviation on the sections analyzed. Right, the percentages of the different types of MKs, including pycnotic cells, were calculated on sections from saline- and DT-treated mice. (**C**) Upper panels, representative images of a stage III MK from untreated mice, left whole cell, right enlarge field showing the presence of α and δ granules and mitochondria (m). Lower panels depict a typical MK from DT-treated mice with a pycnotic nucleus (left panel) and swollen mitochondria (sm)(lower right panel). (**D**) Representative TEM images of bone marrow sections from DT-treated mice on day 8. Note the accumulation of MKs (1), the presence of whole MKs and large fragments of MKs (2) and the presence of proPLTs (arrows), PLTs (arrow heads) (3) and retPLTs (4) in a bone marrow vessel.

Mice transgenic for Cre-inducible diphtheria toxin receptor (DTR) [13] and Cre recombinase under the control of the MK-specific PF4 promoter [14] received DT for 4 days. Profound thrombocytopenia developed during the following days. In general, PLT counts reached a nadir three days later, i.e. on day 7 (22 +/- 17 10^3^ PLTs/μL, n = 20) and rebounded to 250–800 10^3^ cells/μL on day 8 (Fig 1A). In control animals receiving saline, PLT counts were stable (1110 +/- 163 10^3^ PLTs/μL, n = 5).

Since the life span of mouse PLTs is about 5 days [15], this time course indicated that DT killed maturing MKs, while PLT rebound occurred after the restoration of full MK differentiation. This hypothesis was confirmed by TEM analysis of bone marrow from saline- or DT-treated animals, one day after the last dose of DT, i.e on day 5. DT treatment resulted in a two-fold reduction in MK density (Fig 1B, left panel). Classically, four stages of MK differentiation may be identified by TEM, the stages III and IV characterizing PLT-producing MKs [16]. In control animals, the relative frequencies of the different types of MKs increased with maturation, except for the less frequent end-stage (type IV) cells (Fig 1B, right panel, a typical stage III MK is shown in Fig 1C in the first row). In contrast, in DT-treated animals, 24 h after the last dose of DT (on day 5), most of the MKs were viable stage I cells or dead/dying cells displaying a pycnotic nucleus, swollen mitochondria and clear cytoplasm (Fig 1B, right panel, a pycnotic MK is shown in Fig 1C in the second row). Remarkably, on day 8 the bone marrow of DT-treated mice presented evidence of increased megakaryopoiesis, as indicated by the higher MK density (Figs 1D1 and S1), as previously reported [17,18]. Large MK fragments and whole MKs were detected in the bone marrow sinusoids (Fig 1D1 and 1D2), as likewise prePLTs and RER-containing PLTs (Fig 1D3 and 1D4, respectively).

Thus, administration of DT induced the ablation of maturing MKs, blocking PLT generation and resulting in progressive thrombocytopenia. Four days after the cessation of DT treatment (on day 8), megakaryopoiesis and thrombopoiesis were dramatically enhanced.

### Transiently synchronized thrombopoiesis produces a burst of reticulated PLTs

**Fig 2:**
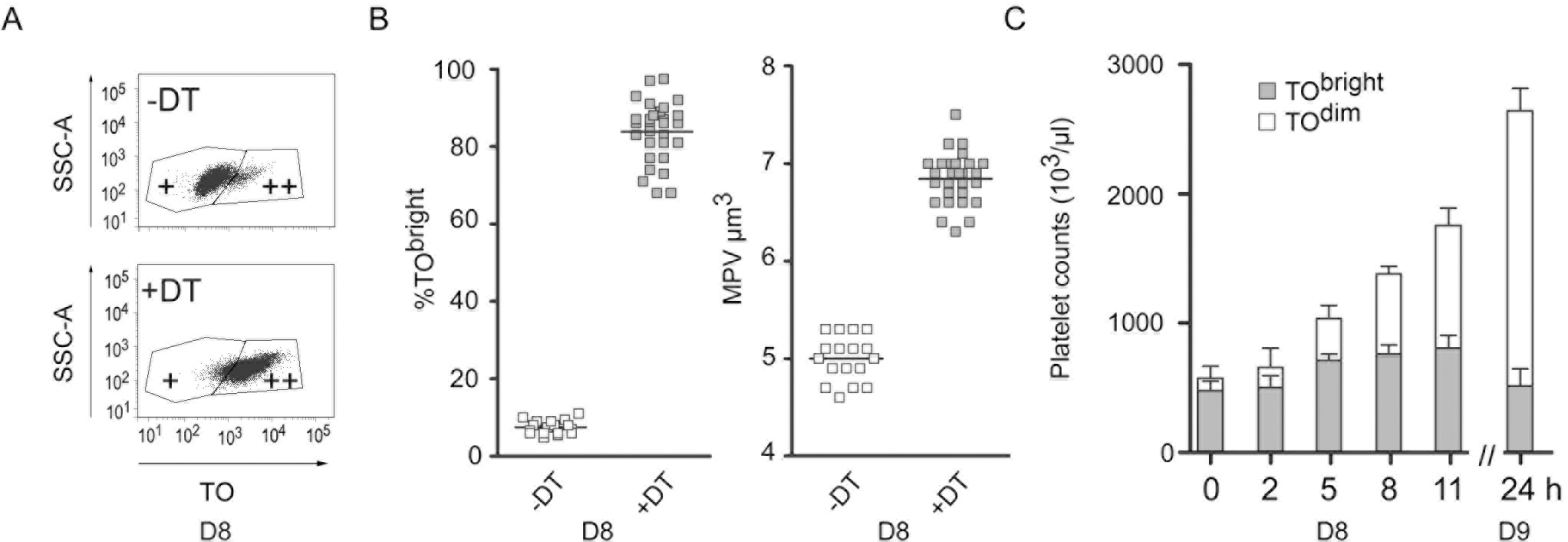
Inversion of the TO^bright^ and TO^dim^ PLT populations. F1 iDTR x PF4-Cre mice were treated as described in Fig 1. On day 8, PLTs were stained with TO and an A647-conjugated anti-GP1bβ mAb and analyzed by FC. (**A**) Two regions, corresponding to TO^bright^ (++) and TO^dim^ (+) PLTs, could be distinguished. (**B**) PLTs were labeled with TO and analyzed by FC to deduce the proportion of TO^bright^ cells (left). The mean PLT volume was determined using a hematology analyzer (right). T-tests indicated a p value of <0.0001 when comparing day 7 and day 8 parameters. (**C**) In a separate experiment, blood samples were drawn on day 8 (0, 2, 5, 8 and 11 h) and day 9 (24 h). Total PLT counts were obtained using a cell counter and the percentage of TO^bright^ PLTs was determined by FC analysis using gates as defined in S3A Fig. The TO^bright^ and TO^dim^ PLT counts were subsequently deduced (n = 5).

This series of events provided the opportunity of analyzing young PLTs in terms of their characteristics and fate. Young PLTs can be distinguished by FC, on the basis of their fluorescence after labeling with TO, an RNA dye. At the beginning of day 8, in control mice, two markedly different TO^bright^ and TO^dim^ PLT populations could be identified (Fig 2A), 7.5 +/- 1.7% (n = 17) of these cells being TO^bright^. Using the same FC analysis strategy for DT-treated animals, this percentage typically exceeded 75–80% (83.8 +/- 7.8, n = 27) (Fig 2B, left panel). The mean PLT volume in DT-treated mice was larger than in control animals (6.8 +/- 0.26 vs 5 +/- 0.23 fL; n = 27 or 17, respectively, p<0.0001) (Fig 2B, right panel). On day 8, TEM analyses of PLTs from DT-treated mice revealed the presence of RER membranes and Golgi sacs, confirming that in contrast to control PLTs, they were reticulated (S2 Fig).

The kinetics of PLT rebound were then followed during day 8. TO^bright^ PLT counts reached their highest levels between time points 8 and 11 h, while TO^dim^ PLT counts already started rising at 5 h, to become the major population between 11 and 24 h (n = 5). PLT counts exceeded twice the normal value when considering the 24 h time point (Fig 2C).

This temporary inversion of the TO^dim^ and reticulated TO^bright^ PLT populations facilitates the handling of retPLTs. Since the time interval during which most of the PLTs are TO^bright^ is narrow, the thrombopoiesis may be considered to be transiently “synchronized”.

### TO labeling can be used to qualitatively analyze the RNA content of mouse PLTs

TO is a fluorescent probe for RNA molecules often used for FC studies of young PLTs. Nevertheless, since the nucleotides in PLT granules can significantly contribute to the fluorescence of TO-stained human PLTs [19], preliminary control experiments were performed with PLTs from saline- or DT-treated mice.

Firstly, PLTs were stained as described in the methods section (labeled with TO and the anti-Gp1bβ mAb RAM1 and fixed with paraformaldehyde, standard staining), or first fixed and permeabilized, treated or not with RNase and then stained with TO and RAM1. FC analysis was done using gates adapted to the staining conditions (fixed *vs* fixed and permeabilized), as detailed in S3A Fig. Under standard staining conditions, 8% of the PLTs from saline-treated animals were TO^bright^ (upper row), vs 82% for DT-treated animals (lower row). Fixation and permeabilization resulted in a decrease in their mean fluorescence intensity, in agreement with the view that nucleotides contribute to the fluorescence of TO-stained PLTs. Further incubation with RNase did not significantly modify the fluorescence of the TO^dim^ cells. In contrast, the fluorescence of TO^bright^ PLTs was reduced to that of TO^dim^ PLTs. Only a minority of PLTs from DT-treated mice remained TO^bright^ (9% of PLTs), but their mean fluorescence intensity was reduced. These experiments demonstrated that the TO fluorescence is an appropriate qualitative indicator of the presence of RNA molecules in TO^bright^ populations.

Secondly, as some of the experiments described used *in vitro* incubations which could be accompanied by degranulation processes, we looked at how thrombin-induced complete degranulation interfered with the analysis of PLTs (S3B Fig). Two types of gate were employed to analyze thrombin-activated PLTs, one optimized for resting control PLTs (first two rows) and used in all other experiments in this paper, the other optimized for activated PLTs (lower row). When activated cells were analyzed, use of the latter gate conserved the ratios of TO^dim^ and TO^bright^ PLTs (lower left histogram). In contrast, a moderate decrease in the percentage of TO^bright^ PLTs was observed if the gates defined for resting PLTs were used (85.1 ± 1.5 vs 74.4 ± 2.5% TO^bright^ PLTs for cells from DT-treated animals, 5.8 ± 0.3 vs 3.5 ± 0.2% TO^bright^ PLTs for cells from saline-treated mice, activated vs resting PLT gates, respectively, n = 3). These experiments confirmed that the fluorescence intensity of TO^dim^ and TO^bright^ PLTs was reduced after degranulation; nevertheless, the percentage of TO^bright^ PLTs was moderately underestimated when the gates defined for resting PLTs were used.

### Fate of TO^bright^ PLTs *in vitro* and *in vivo*: TO fluorescence and *in vivo* lifespan

**Fig 3:**
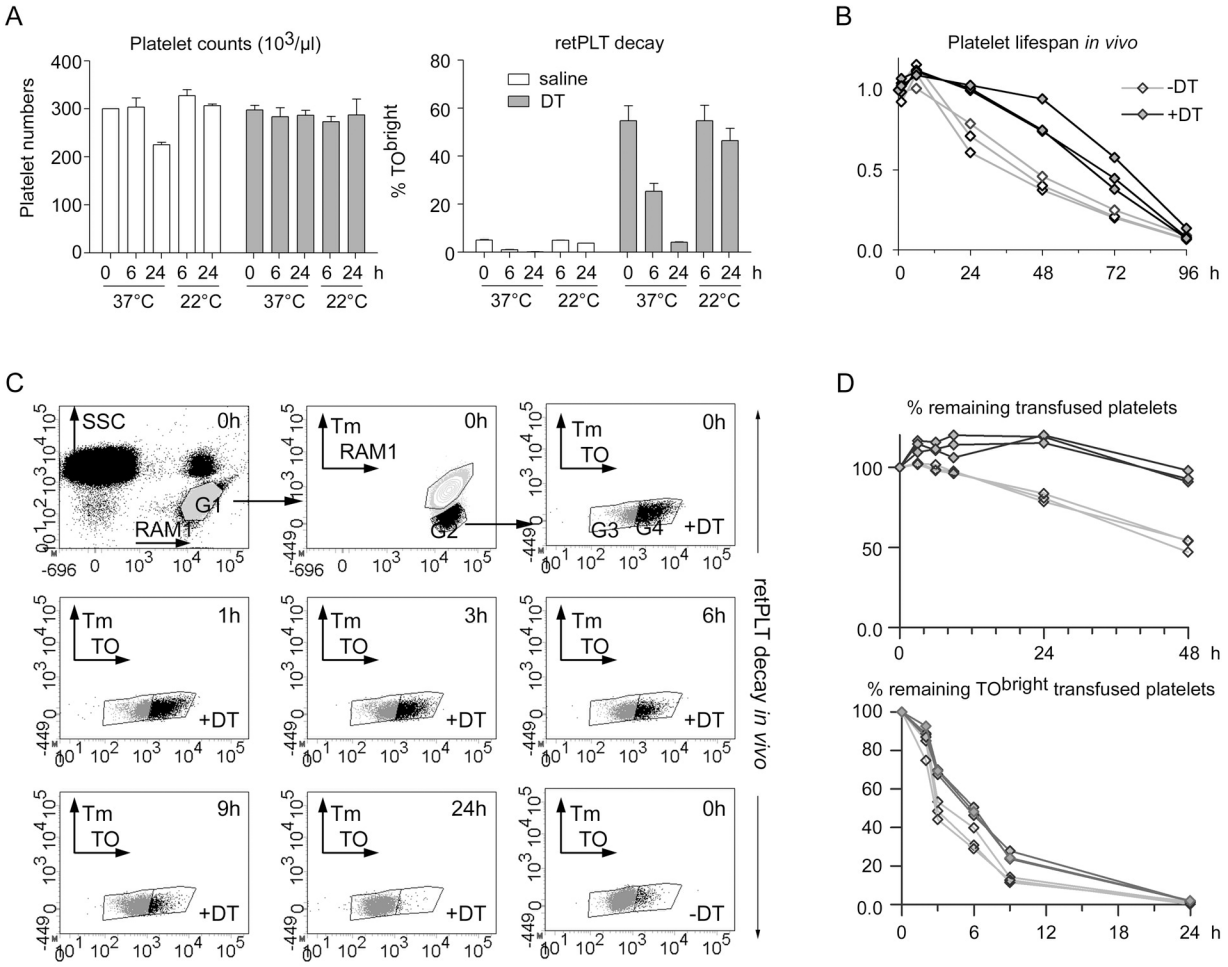
Life span of retPLTs *in vitro* and *in vivo*. (A) On day 8, washed PLTs were prepared and incubated for the indicated times at 22°C or 37°C in Tyrode’s albumin/DMEM medium. At each time point, PLT counts were recorded (left panel) and cell samples were stained with TO and analyzed by FC to determine the percentage of TO^bright^ PLTs (retPLTs) (right panel) (saline- and DT-treated animals, white and grey bars, respectively) (n = 3). (B) On day 8, pooled washed PLTs were prepared from 3 saline- or DT-treated F1 iDTR x PF4-Cre mice and injected into 3 mice expressing EGFP in all cell types. Blood samples obtained 15 min, 1 h 15, 6 h 15 and 24 to 96 h after PLT transfusion (respectively time points 0 h, 1 h, 3 h, etc.) were stained with Alexa647-conjugated RAM1 and analyzed by FC. As also depicted in (C), the RAM1^+^/SSC^dim^ gate (G1) corresponding to all single PLT events was defined and then the RAM1^+^/EGFP^-^ transfused PLTs (G2). The percentage of transfused PLTs at a given time (ratio of the number of events in G2 to that in G1) was normalized to that at time 0 to deduce the percentages of remaining transfused PLTs. (C) On day 8, pools of washed PLTs from saline (-DT) or DT- (+DT) treated mice were transfused in Tomato-expressing mice and their fate was followed for 48h. Blood samples obtained 15 min, 1 h 15, 3 h 15, 6 h 15, 9 h 15, 24, and 48h after PLT transfusion were stained with TO and Alexa647-conjugated RAM1 and analyzed by FC. The first row depicts the gating strategy used to analyze the fate of transfused non-fluorescent PLTs: RAM1^+^/SSC^dim^ gate (G1) corresponding to all single PLT events was defined and then the RAM1^+^/Tm^-^ transfused PLTs (G2) and the Ram1+/ TO^dim^ (G3) and TO^bright^ (G4) transfused PLTs were successively selected. Lower dot plots represent the distribution of remaining transfused PLTs at different times. The G3 and G4 gates were defined, based on the profile of control PLTs from saline-treated mice at time 0 (lower right dot plot). (D) Upper panel, the percentage of remaining transfused PLTs at a given time (ratio of the number of events in G2 to that in G1) was normalized to that at time 0. Lower panel, the ratio of the number of events in G4 to that in G2 provided the percentage of remaining TO^bright^ cells among transfused PLTs.

The dynamics of the TO^dim^ and TO^bright^ PLT populations (Fig 2C) suggested that within less than 12 h, most TO^bright^ PLTs became TO^dim^ (compare 0 h vs 11 h and 11 h vs 24 h). To better analyze these dynamics, washed PLTs were prepared on day 8 from saline- or DT-treated mice and incubated *ex vivo* for 6 or 24 h at 22 or 37°C. During the incubation, total numbers of PLTs remained stable (Fig 3A, left panel). FC analysis of PLTs labeled with JONA-PE, an anti-activated GPIIbIIIa mAb, indicated that the vast majority of them (93.4 ± 0.8% vs 96.4 ± 0.2% for PLTs from saline- or DT-treated animals, respectively, n = 3) remained non-activated after 6 h, as also a majority of the cells after 24 h at 37°C (69.5 ± 0.4% *vs* 89.4 ± 0.8% for PLTs from saline- or DT-treated animals, respectively) (S4A Fig). Electron microscopy revealed that most if not all of the PLTs incubated at 37°C for 24 h were discoid and contained alpha and dense granules, whereas only a very small number of necrotic/apoptotic PLTs was observed. Thus, the major signs of activation were missing (S4B Fig, data only shown for PLTs from DT-treated mice). FC analysis of TO-stained PLTs confirmed the presence of a large majority of TO^bright^ cells after DT treatment, indicated that the percentage of TO^bright^ PLTs was stable at 22°C but decreased by a factor of two within the first 6 h and, was below 5% after 24 h at 37°C (4.16 +/- 0.20%, n = 3; Fig 3A, right panel).

To confirm that these *in vitro* observations were representative of *in vivo* physiology, pools of washed PLTs from saline or DT-treated mice were prepared, and transfused into recipient mice expressing EGFP or Tomato fluorescent protein in all their cells. Since PLTs from DT-treated animals are young, we anticipated that their lifespan would be longer than that of PLTs from saline-treated control mice. Indeed, 48 h after transfusion into EGFP^+^ mice, the count of PLTs from saline-treated animals decreased by 50%, whereas that of PLTs from DT-treated animals was stable (Fig 3B). Two way ANOVA followed by a Bonferroni post hoc test comparing PLTs from saline- and DT-treated animals revealed that the differences in the numbers of remaining transfused retPLTs vs control PLTs displayed a p value of <0.001 at times 24, 48 and 72 h. The half-life of transfused retPLTs was between 48 and 72 h, in agreement with the estimated 5 day lifespan of PLTs in mice [20], confirming the good quality of the transfused cells. These observations also highlight the potential advantages of retPLTs for transfusion.

The fate of the TO^bright^ phenotype was then investigated *in vivo*. Pools of washed PLTs from saline or DT-treated mice were transfused into Tomato-positive mice and their fate was followed for 48 h. Blood samples were drawn at different times and the PLTs were counted and labeled with TO before analysis by FC (Fig 3C). At time 0 (15 min after transfusion), the ratio of Tomato-negative transfused PLTs to Tomato-positive recipient PLTs was 4–6%. As observed in transfusion experiments in EGFP^+^ mice, the numbers of transfused PLTs from DT-treated animals remained fairly stable for 48 h, while the numbers of transfused control PLTs decreased by 50% within this period. A two way ANOVA followed with a Bonferroni post hoc test comparing PLTs from saline and DT-treated animals revealed that the differences in the percentages of remaining transfused PLTs at times 6, 9, 24 and 48 h (Fig 3D, upper panel) displayed p values of < 0.05, 0.01, 0.001 and 0.001, respectively. When the TO^bright^ PLTs were analyzed, a decrease of their relative number could be noticed as soon as 3 h after transfusion, while only a few remained 24 h later (Fig 3D, lower panel). One may also note that the percentage of TO^bright^ PLTs from saline-treated animals appeared to decrease more rapidly; two way ANOVA showed a p value 0.001 for the differences between saline- and DT-treated animals at times 3, 6 and 9 h.

Thus, in both *ex viv*o incubations at 37°C and *in vivo* experiments, the TO^bright^ phenotype was lost within 6 h in about 50% of PLTs, and was almost completely lost in less than 24 h.

### Comparison of the RNA contents of control and TO^bright^ PLTs

Under our experimental conditions, TO fluorescence qualitatively indicated the presence of RNA in PLTs. We thus checked how the mean fluorescence intensity of TO-labeled PLTs was quantitatively related to their “RNA content”, here corresponding to Trizol-extracted RNA purified on silica matrix, thus excluding small RNAs. On day 8 of the protocol, erythrocyte- and leukocyte-depleted PLTs were prepared from mice receiving saline (control) or DT. FC analysis of 10^6^ PLTs showed that the content of residual erythrocytes and leukocytes was less than 2/10^6^ PLTs (S5 Fig).

Purified RNA molecules were checked by capillary electrophoresis on a Bioanalyzer. This instrument provides two indicators of the RNA quality, the RNA integrity number (RIN) and the ratio of the amounts of 28S and 18S ribosomal RNA (rRNA). The algorithm for RIN determination was developed by applying adaptive learning tools to analyses of nucleated mammalian cells. In this general situation, RIN is the preferred measure of RNA quality, due to its better reproducibility [21]. A RIN value of 10 is optimal and is usually obtained for RNA extracted from freshly isolated nucleated cells as in the case of *in vitro* differentiated MKs. RIN values of RNAs from retPLTs were above 9 (S6A Fig), whereas this parameter was lower but could exceed 8 for PLTs of saline-treated animals (not shown). The profiles of the RNA from control PLTs were characterized by the presence of multiple marked peaks, contrary to what was observed for nucleated cells and retPLTs (S6A Fig), making the interpretation of the RIN number delicate for PLTs. Consequently, we preferred to use the 28S/18S ratio as quality indicator of purified PLT RNA. Higher values of 28S/18S ratio (>1.3) were correlated to retPLTs percentages above 90% (S6B and S6C Fig), whereas this ratio was in general about 0.9 for control PLTs (S6C Fig). The ratio of purified RNA to the number of freshly isolated PLTs could reach 20 to 40 fg/PLT for preparations displaying 80–90% TO^bright^, much more than for PLTs from untreated animals (0.6–0.9 fg/PLT), containing only 5–10% TO^bright^ PLTs (S6D Fig). Since under normal conditions less than 10% of PLTs are TO^bright^, these data provide quantitative confirmation of previous qualitative estimations of the very low RNA content of TO^dim^ PLTs deduced from FC analyses of TO-labeled PLTs [22].

## Decay of rRNA in retPLTs

**Fig 4:**
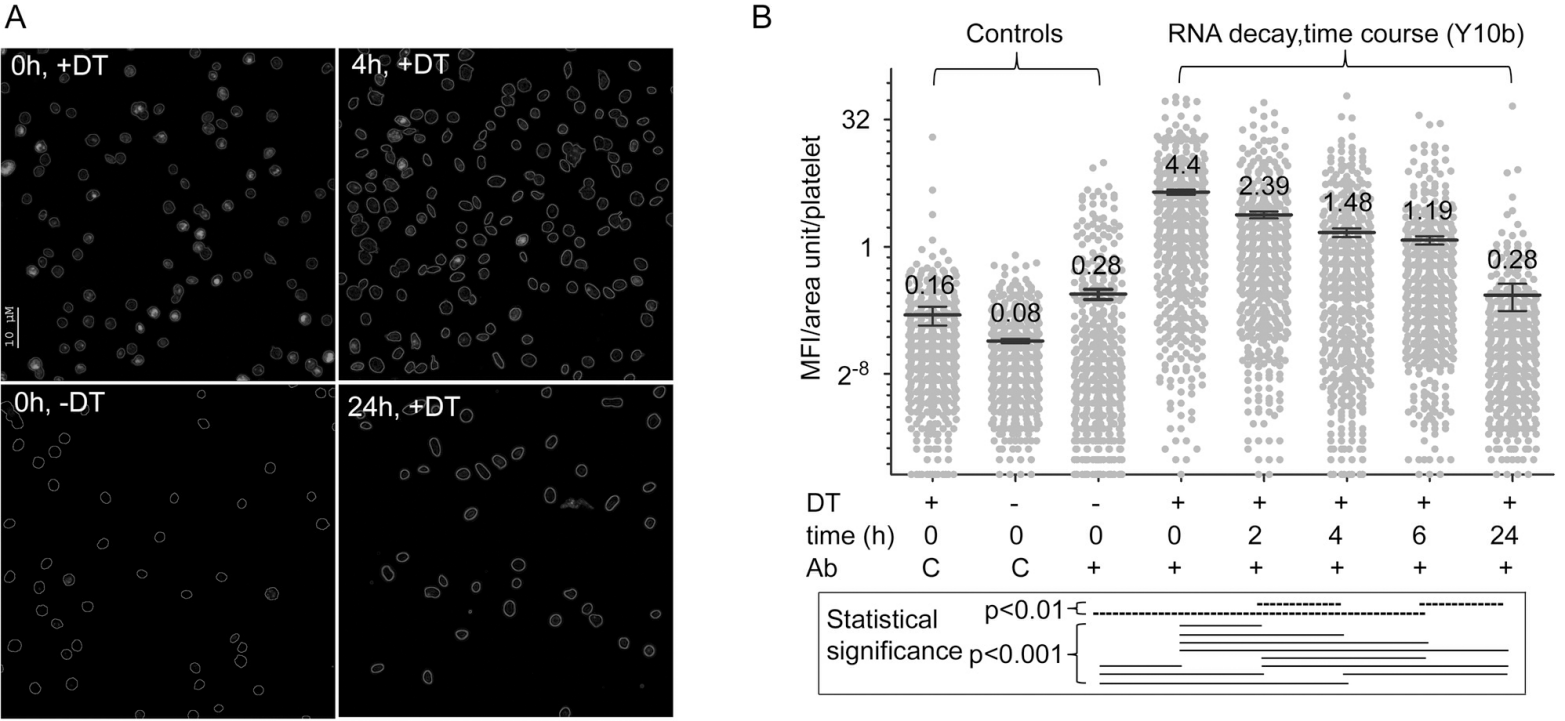
Fate of rRNAs. On day 8, washed PLTs from DT- or saline-treated mice were resuspended in Tyrode’s albumin/DMEM medium and incubated at 37°C for different periods of time. (A) PLTs were incubated for 0, 2, 4, 6 and 24 h. The percentage of TO^bright^ PLTs, determined at 0, 6 and 24 h, was 82, 33 and 2%, respectively. At each time points, PLT samples were fixed and permeabilized, stained with the anti-rRNA mAb Y10b and A647-conjugated anti-mouse IgGs, counterstained with an A488-conjugated anti-GP1bβ mAb and analyzed by confocal microscopy. PLT outlines, determined by Photoshop analysis of anti-GP1bβ staining, are depicted. Representative micrographs of analyzed cells from DT-treated animals (+DT) at times 0, 4 and 24 h and from saline-treated animals (-DT) at time 0 are shown. (B) The mean fluorescence intensity per unit area in each PLT stained with Y10b (+) or an isotype-matched control antibody (C) (y-axis, log2 scale) was calculated as described in the methods; 500 to 550 PLTs from DT-treated mice (+) or saline-treated mice (-) were analyzed for each condition. A one way ANOVA analysis followed by a Bonferroni post-hoc test comparing each pair of conditions gave p values of <0.001 (continuous bars) or <0.01 (dashed bars). Other combinations of Y10b stained samples were not statistically different.

Altogether, these results suggested that retPLTs lose their TO^bright^ fluorescence because their RNA content decreased. To confirm this hypothesis, washed retPLTs were incubated *in vitro* at 37°C for different periods of time. At each time point, the PLT counts were controlled and PLTs were stained with TO and analyzed by FC, or alternatively fixed, permeabilized, immunolabeled with the anti-rRNA monoclonal antibody Y10b [23,24] and analyzed by confocal microscopy [25]. FC analysis showed that at times 0, 6 and 24 h, 80, 33 and 2% of the PLTs were TO^bright^, respectively. In immunofluorescence microscopy experiments, Y10b did not label retPLTs treated with RNase, confirming its specificity (S3C Fig). At time 0, PLTs from DT-treated mice were more strongly stained by Y10b than PLTs from mice receiving saline (Fig 4A, left panels), while Y10b labeling of the retPLTs decreased within a few hours (e.g. at 4 and 24 h, Fig 4A, right panels). Analysis of 500 cells for each condition revealed that the fluorescence intensity of retPLTs diminished fourfold within 6 h (Fig 4B) and after 24 h at 37°C, it dropped to that of PLTs from animals receiving saline. Since the mAb Y10b recognizes 5.8S, 18S and 28S rRNA [24] and 5.8S rRNA controls ribosome translocation [26], this decreased fluorescence strongly suggested that intact and therefore probably functional ribosomes decayed during the period of analysis.

### RNA content and integrity decrease in PLTs incubated *ex vivo* at physiological temperature

**Fig 5:**
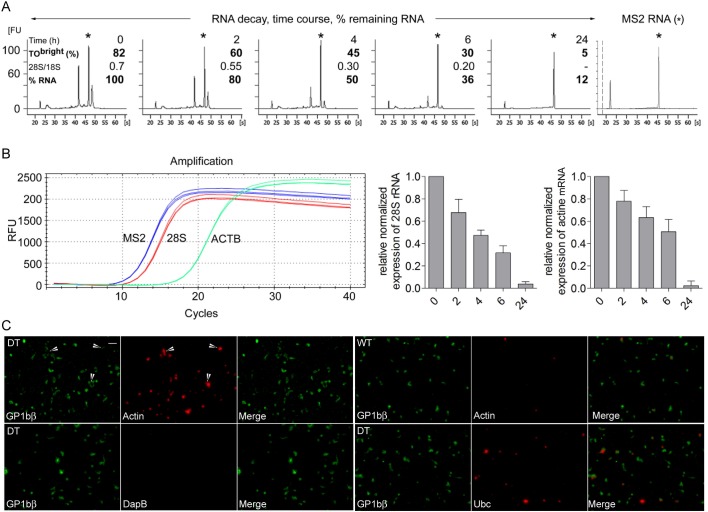
Decay of the quality of PLT RNA. In another series of experiments, washed PLTs from DT-treated mice were resuspended in Tyrode’s albumin/DMEM medium and incubated at 37°C for 0, 2, 4, 6 and 24h. At the end of each time interval the percentage of TO^bright^ PLTs was determined by FC, after which PLT RNA was extracted in Trizol reagent containing MS2 genomic RNA (see methods) (n = 4). (A) The profiles of extracted RNAs were analyzed on a Bioanalyzer; a representative series of profiles is shown. Y-axis scales (fluorescence units) are identical for all the electropherograms (20 FU per subdivision). The asterisks indicate the position of MS2 RNA and the right electropherogram corresponds to pure MS2 RNA. First number, time; second number, percentage of TO^bright^ PLTs; third number, 28S/18S RNA ratio; fourth number, percentage of remaining PLT RNA. (B) The decays of rRNA and beta actin mRNA were analyzed by RT-qPCR (see details in method section). Representative amplification curves for MS2, 28S rRNA and beta actin cDNAs at time 0 are shown (1/50 diluted samples, triplicates). No specific amplification products were generated in control experiments without reverse transcriptase (not shown). The relative normalized expression of 28S rRNA and of beta actin mRNA relatively to time 0 were calculated as described in the method section. Histograms represent means and SEM from the 4 independent experiments. C) The presence of beta actin and ubiquitin C transcripts in freshly isolated control and reticulated PLTs was assessed using RNAscope technique. The stringency of the conditions was checked using *B subtilis*-specific DapB mRNA probes. Hybridized probes were revealed using alkaline phosphatase and HD-assay RED reagent. PLTs were then counterstained with A488-conjugated anti-GP1bβ mAb and DAPI. Upper panels, retPLTs (left, DT) and control PLTs (right, WT) labelled with actin mRNA-specific probes. The masking effect of RED reagent-derived large precipitates on anti-GP1bβ staining is illustrated by arrows. Lower left panel, retPLTs labelled with negative control *DapB* probes. Lower right panel, retPLTs were stained with Ubiquitin C-specific probes. DAPI staining is not shown because no cells were labelled in the chosen views. Scale bar: 10 μm.

The above observations were confirmed by direct biochemical analysis of the fate of the PLT RNA. Leukocyte- and erythrocyte-depleted retPLTs were prepared and incubated *in vitro* at 37°C for different periods of time. At each time point, the PLTs counts were controlled and the cells were stained with TO and analyzed by FC. Total RNA was then extracted with Trizol, without adsorption step on silica matrix, in the presence of defined amounts of bacteriophage MS2 genomic RNA, as an internal control of RNA extraction. The amounts of MS2 RNA recovered were similar in all preparations, indicating similar yields for all extractions and thus that no significant RNA degradation occurred during the extraction procedure. In contrast, the PLT rRNA peaks strongly diminished within the first 6 h and were no longer detected after 24 h (Fig 5A). The quantity of PLT RNA, deduced from the electropherograms and normalized to the quantity of MS2 RNA recovered in each sample, was reduced by a factor of 2 after 4 h of incubation at 37°C. During this period of time, the 28S/18S ratio decreased twofold, here again indicating that the activity of the remaining ribosomes could be strongly compromised.

The decays of 28S rRNA and of beta actin mRNA molecules in PLTs incubated *in vitro* at 37°C were also analyzed by reverse transcriptase followed by real time quantitative PCR (RT-qPCR). MS2 RNA added as internal control of RNA extractions was used to normalize the data. These experiments not only confirmed the rapid decay of rRNA, but also indicated that mRNAs such as beta actin transcripts also decay with a rather similar kinetics (Fig 5B). In control experiments without reverse transcriptase, no specific amplification products were generated (data not shown). Of note, at time 0, the ratio of the copy numbers of reverse transcribed beta actin and 28S rRNA molecules deduced from these experiments was 0.02 ±0.007 (n = 5).

We then compared the presence of transcripts in retPLTs and control ones using RNAscope fluorescence *in situ* hybridization technique. PLT rich plasma were prepared from saline or DT-treated animals; FC analysis confirmed that more than 98% of the cells were PLTs, of which 6 and 93% were reticulated, respectively (not shown). PLTs were processed and labeled with beta actin- or ubiquitin C-specific probes, or *B*. *subtilis DapB* negative control ones. Hybridized probes were revealed by alkaline phosphatase and the fluorescent HD-assay RED reagent, which forms a precipitate visible using bright field or fluorescence microscopy. The samples were also counter-stained with Alexa 488-conjugated anti-GP1bβ mAb (Fig 5C) and DAPI (DAPI^+^ cells were rare and absent in Fig 5C views). The irrelevant *B subtilis DapB* probes did not significantly stain PLTs (lower left panels), demonstrating the stringency of the experimental conditions. When retPLTs were labelled with actin or ubiquitin C mRNA probes, cells brightly stained with the HD-assay RED reagent were often poorly labelled with the anti-GP1bβ mAb (see representative events indicated by arrows on the Fig 5C, upper left panels). Since these cells were not stained with DAPI (not shown) and because 98% of the cells in the treated samples were PLTs, the weak anti-GP1bβ labelling likely results from a masking effect resulting from the precipitation of the reaction products. Analysis of several micrographs revealed that about 8% of the control PLTs (25 out of 320 GP1bβ^+^ cells) and 80%f retPLTs (230 out of 291 GP1bβ^+^ cells) were stained with the beta actin probe (Fig 5C, upper panels). About 22% of the retPLTs (46 out of 204 GP1bβ^+^ cells) were labelled by anti-ubiquitin C mRNA probes (representative micrographs, lower right panels), whereas control PLTs were virtually not stained (not shown). The pattern of beta actin mRNA distribution is in agreement with the quantitative difference of RNA content between reticulated and control PLTs established with RT-qPCR assays and flow cytometric assays.

### Translation rapidly decreases in PLTs incubated *ex vivo* at physiological temperature

**Fig 6:**
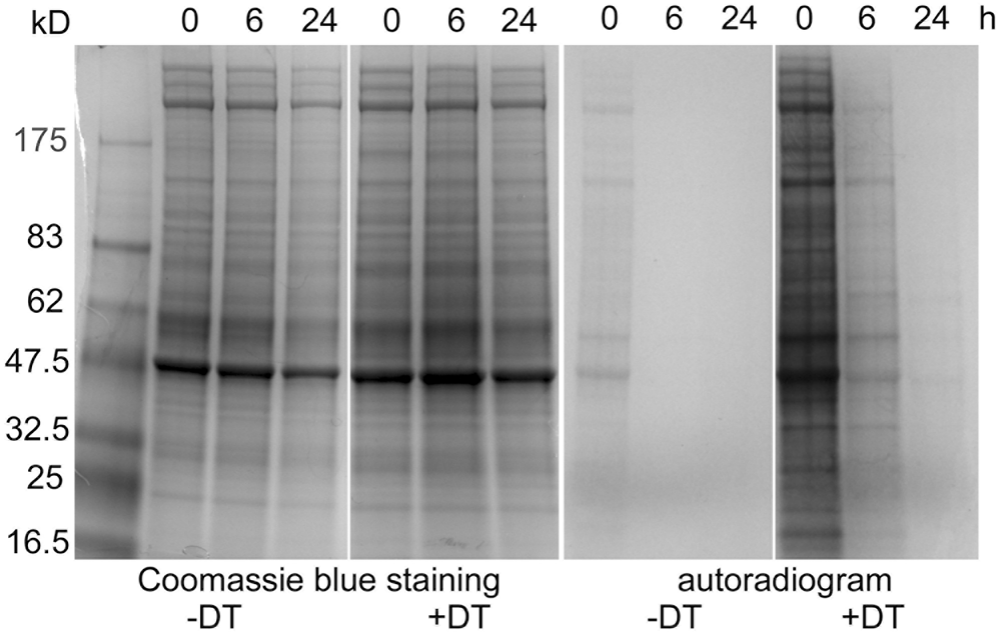
Decay of translation in PLTs. Washed PLTs were prepared from saline- (-DT) or DT-treated mice (+DT), incubated for 0, 6 and 24h at 37°C and metabolically labeled with ^35^S methionine and cysteine for 30 min as described in the method sections. PLT lysates (25μg/lane) were analyzed by SDS-PAGE under reducing conditions, Coomassie blue-stained (left panels), or autoradiographed for 4 days (right panels).

These findings implied that translation in PLTs should rapidly decay. To check this conclusion, washed and leukocyte- and erythrocyte-depleted PLTs were prepared from saline- and DT-treated mice and incubated or not for different periods of time at 37°C. Proteins were metabolically labeled with ^35^S methionine and cysteine for 30 min and then analyzed by SDS-PAGE. Coomassie blue staining confirmed that similar amount of proteins were analyzed in all conditions. Autoradiography demonstrated that constitutive translation was much more efficient in reticulated than in freshly isolated control PLTs. In retPLTs, it strongly decreased within the first 6 h of incubation and was very weak after 24 h, while at these times it was not observable in control cells (Fig 6).

### RNA content of human PLTs in transfusion bags

**Fig 7:**
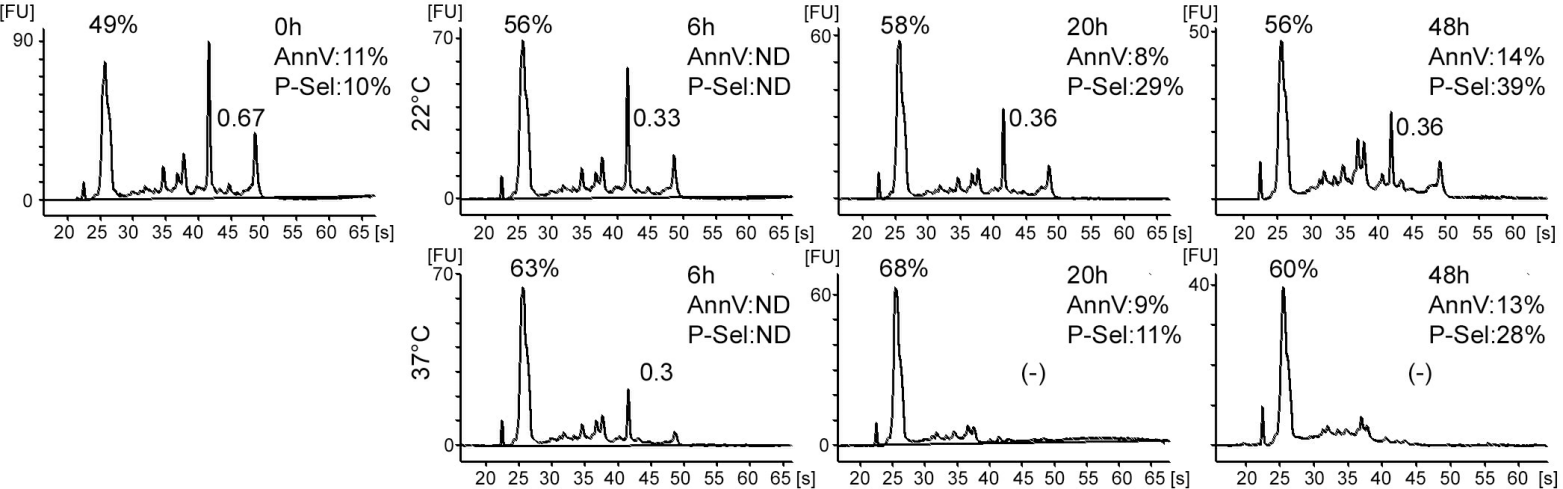
Fate of human PLT RNAs in transfusion bags. Transfusion bags were incubated and sampled at 22°C or 37°C for 0, 6, 20 or 48 h. PLT activation was checked by FC after staining with FITC-conjugated annexin V or anti-P-selectin mAb. Total RNA was extracted and analyzed on a Bioanalyzer 2100 and electropherograms of the extracted RNA are shown. The 28S/18S RNA ratios are indicated between the rRNA peaks and the percentages of small RNAs are shown above the small RNA peaks. AnnV and P-Sel, percentage of annexin V- and P-selectin-positive PLTs (ND, not determined).

Finally, we examined the RNA content of human PLTs in bags of PLT concentrates prepared for transfusion, which represent pools of 5 donors, so that possible inter-individual variability is smoothed. These bags were kept for different periods of time ranging to up to 48 h, at 22°C, their storage temperature before transfusion, or 37°C. The pH and pO_2_ remained stable during this time frame (pH_22°C_ 7.2–7.3, pH_37°C_ 7.0–7.3; pO_2_117-150 mm Hg at both temperatures). FC analysis of PLTs labeled with annexin V showed no major differences at any time point or temperature (8%-14% positive PLTs in the analyzed samples), while as usually observed in storage conditions, P-Selectin was exposed on the PLTs after incubation (33 and 40% at 22°C, 11 and 28% positive PLTs at 37°C after 20 and 48 h, respectively). At each time point, RNA was extracted with Trizol reagent and analyzed (Fig 7). The mean RNA content of the PLTs in newly prepared bags was 0.6 fg/PLT, while the presence of large amounts of small RNA molecules was a characteristic feature; these represented about 50% at time 0 and more at later time points. Small RNAs were still present after 48 h, compatible with their abundance at time 0 in a mix of 0 to 10 day old blood PLTs. The rRNA peak heights later decreased relative to the peaks of small RNAs, indicating degradation of rRNA, a property which was amplified at 37°C. This degradation was confirmed by the evolution of the 28S/18S ratio, from just below 0.7 at time 0 to approximately 0.3 at 6 h, whether the cells were incubated at 22°C or at 37°C. After 48 h at 22°C, rRNAs were still present, whereas at 37°C these molecules were already no longer detectable after 20 h.

Thus, in human PLTs kept in transfusion bags, the life span of rRNA was less than one day at 37°C. However, rRNA was better preserved at the storage temperature.

## Discussion

It is well known that PLTs contain small amounts of RNA. This RNA is mostly present in newly generated PLTs, said reticulated due to the presence of the RER translation site. Constitutive translation in all PLTs has been documented; on one hand it is more active in young PLTs [27], but on the other hand, leaving aside PLT subpopulations, it appears to mainly occur in mitochondria [28]. We here revisited this topic by investigating the changes in the RNA content (defined as the ratio of the amount of extracted RNA molecules to the number of PLTs, or the yield of extracted RNA per PLT, or by TO fluorescence) in PLTs, freshly isolated or aging *ex vivo* or *in vivo*. In addition we analyzed the *ex vivo* decay of 28S rRNA and beta actin mRNA by RT-qPCR and, we compared the distribution of beta actin transcripts in freshly isolated control and retPLTs using RNAscope fluorescence *in situ* hybridization technique. The consequences for constitutive protein synthesis were then evaluated.

This was made possible by inducing a transient state characterized by the presence of a vast majority of young PLTs. After administration of DT in mice expressing its receptor in MKs, mostly stage I MKs (characterized by a horseshoe-shaped nucleus and the absence of specific granules [16]) and pycnotic MKs were observed in the bone marrow. Thus, in DT-treated mice, mature MKs were absent and consequently, thrombopoiesis was impaired. Recently, this model of MK ablation has been used to demonstrate a role of MKs in the homeostatic regulation of stem cell populations; in particular MK ablation appeared to result in the loss of their quiescence [17,18].

Four days after cessation of the treatment, the MK density in the bone marrow was about 5 fold that in control mice, indicating that the treatment had induced the accumulation of MK progenitors. At that time, thrombopoiesis was very active, as indicated by electron microscopy images of bone morrow and kinetics of PLT counts, so a spectacular transient inversion of the proportion of young PLTs in mice was noticed, as assessed by FC after staining of PLTs with TO (TO^bright^ PLTs). The mean volume of these PLTs was significantly increased, as is seen in humans when thrombopoiesis is enhanced, for example in different forms of thrombocytopenia [10]. TEM showed that these cells contained RER and Golgi compartments, which are rarely observed in PLTs under normal conditions, but have been described in the context of idiopathic thrombocytopenic purpura [12].

*Ex vivo* experiments (time course analyses of washed PLTs incubated at 37°C for different periods) and *in vivo* assays (time course analyses of PLT subsets during experimental thrombocytosis or after transfusion) allowed analysis of the time-dependent changes in the RNA content of PLTs. These changes were assessed (i) by FC determination of the relative numbers of TO^dim^ and TO^bright^ cells, (ii) by extracting PLT RNA content and calculating the ratio of the amount of extracted RNA to the number of PLTs, by capillary electrophoretic analysis of extracted RNA molecules, (iii) by RT-qPCR analysis of 28S rRNA and beta actin mRNA decay and (iv) by immunofluorescence staining of PLTs with an anti-rRNA antibody. E*x vivo* experiments allowed follow-up of the RNA content of PLTs from a given time point, avoiding the confounding continuous generation of new retPLTs. As discussed below, *in vivo* assays confirmed the validity of the *ex vivo* strategy.

Part of the TO fluorescence of PLTs has been attributed to the binding of TO to nucleotides in dense granules [19]. Control experiments confirmed that RNA molecules were responsible for most of the fluorescence of TO^bright^ PLTs, but did not significantly contribute to that of TO^dim^ PLTs. In addition, we validated our strategy for the FC analysis of PLT subsets by showing that under our conditions, the relative proportions of the TO^dim^ and TO^bright^ subsets were moderately affected by complete PLT degranulation.

In a first series of experiments, we showed that TO^bright^ PLTs from DT-treated animals incubated *ex vivo* at 37°C were twofold less numerous after 6 h and had completely disappeared after 24h. At these times, most of the PLTs from DT-treated animals incubated at 37°C did not express an activated phenotype, as shown by the low percentages of PLTs expressing activated GPIIb-IIIa (<5% after 6h, <10% after 24 h, S4A Fig). Notably, electron microscopy showed that most of the cells displayed no signs of activation. These data strongly suggest that PLT activation does not contribute to a major extent to the decay of fluorescence in retPLTs from DT-treated mice incubated *ex vivo*. In contrast, after 24 h at 37°C, but not at early time points, activated GPIIb-IIIa was expressed on nearly 30% of control PLTs. This property probably reflects the expected presence of a relatively important fraction of 3–5 day old PLTs, which could be more susceptible to senescence processes, most likely due to the decay of anti-apoptotic proteins in aging PLTs [29,30]. In any case, these observations for control PLTs at the 24 h time point do not alter the validity of the conclusions deduced from the analysis of other conditions (i.e., at other time points and in PLTs from DT-treated animals), where activated GPIIb-IIIa was present on only a small fraction of PLTs.

*In vivo*, the counts of transfused PLTs from saline-treated animals decreased by 50% within 48 h, whereas those of PLTs from DT-treated animals remained quite stable for 48 h; this difference also probably reflects the respective ages of the transfused cells. The count of the TO^bright^ population among transfused PLTs decayed with kinetics similar to those observed for retPLTs incubated *in vitro* at 37°C, strongly suggesting that our observations concerning PLTs incubated *in vitro* are biologically relevant. Finally, in DT-treated animals, during the first day of thrombocytosis the continuously generated TO^bright^ PLTs appeared to become TO^dim^ within 12 h (compare time 0 h vs 11 h and 11 h vs 24 h). Thus, all our *in vitro* and *in vivo* observations were concordant and indicated that the numbers of TO^bright^ PLTs decreased with comparable kinetics in the two conditions.

Biochemical analyses confirmed the correlation between the percentage of TO^bright^ PLTs and the estimated mean RNA content of PLT preparations. This correlation was observed in different preparations of freshly isolated, more or less reticulated PLTs (S6 Fig). Ribosomal RNAs disappeared in PLTs incubated *in vitro* at 37°C. According to our *in vitro* data, their half-life appeared be rather short, possibly 4 h, as compared to the values measured in tissues or cultured cells which exceed a few days [31–33]. RT-qPCR assays indicated that mRNA could also rapidly decay in retPLTs, as exemplified with beta-actin mRNA, one of the most abundant protein-encoding transcripts in PLTs. In addition, the RT-qPCR analysis of RNA extracted at time 0 indicated that the relative normalized expression of beta actin mRNAs to 28S rRNA was 0.02 ±0.007, which was substantially higher than for *in vitro* differentiated mouse megakaryocytes (0.003, n = 3, data not shown) and, than their relative numbers in human cell lines (about, 0.001) [34]. Although additional experiments could be necessary to define the actual ratio of the corresponding RNA molecules in PLTs, these data are compatible with the observed over-representation of beta actin sequence reads in RNA-seq experiments [35–37]. The limited presence of beta actin mRNAs in PLTs could be confirmed with the use of RNAscope technology, a powerful method allowing the detection of specific transcripts in individual cells. Indeed, beta actin probes stained the vast majority of retPLTs (80% of them), but in contrast, only a minority of control PLTs (8% of them). These observations reflect at an individual PLT level our results about the RNA content of entire PLT populations from control and DT-treated animals.

The ratio of the amount of extracted RNA to the number of freshly isolated PLTs could reach 20 fg/retPLT, even 40 fg/retPLT when more than 90% of the cells were TO^bright^, but was less than 1 fg/PLT in control PLTs, where only 5–10% of the cells were reticulated. These estimates strongly suggest that non-reticulated PLTs contain on average substantially less than 1 fg/PLT. One may note that, based on the total molecular mass of 18S and 28S rRNA (2.24 10^6^ D), 1 μg of RNA contains less than 2.68 10^11^ 18S and 28S molecules, or in simplistic terms less than 268 copies of rRNA per fg. Our experiments indicate that most of these molecules are detected in retPLTs, which raises the question of whether non-reticulated PLTs are equipped with efficient translation machinery, especially if the translation elongation rate is taken into account. In mouse stem cells, this rate has been estimated to be 5.6 amino acids per second [38], although of course this rate needs to be precisely determined for PLTs. In any case, consistent with our observations, metabolic labeling revealed that in retPLTs, most of the translation activity vanished within 6 h at 37°C, approaching that of freshly isolated PLTs (Fig 6, compare + DT at 6h and—DT at 0h). These observations strongly suggest that under normal conditions, constitutive translation is confined to the minor fraction of retPLTs.

We observed that the RNA content of mouse and human PLTs was more labile at 37°C than at 22°C. This temperature dependence indicates that RNA degradation could be coupled to cellular processes, for example RNautophagy [39,40], microparticle generation or mobilization of RNase from subcellular structures. *A priori*, the *in vitro* generation of microparticles from PLTs would contribute to the concomitant consumption of assembled ribosomes and hence of both types of rRNA, which did not appear to occur to a major extent. The faster disappearance of the 28S rRNA peak would be better explained by RNase-dependent processing.

Several reports have concluded that activation of human or mouse PLTs resulted in IL-1β or Bcl3 synthesis [41–43], while components of the splicing and translational machineries were found to be present and to participate to activation-induced mRNA splicing followed by their translation [44,45]. Moreover, *in situ* RT-PCR experiments followed by hybridization with PCR-generated probes indicated the presence of spliced IL1-β mRNA in all activated platelets [46]. In contrast, another study failed to provide evidence that leukocyte-depleted PLTs release IL-1β [5]. If we take into account our analysis of the RNA content of PLTs and the genetic profiling studies of human PLTs using RNA-seq methods [35,36,47], translation in PLTs can be discussed from another point of view. The values of the normalized expression ratios of genes relative to that of β-actin are available [36,37] or can be deduced from the data provided [35]. These compatible data indicate that 60–90 genes [37] or about 200 genes [35,36] display a ratio of >0.01, in other words, the number of their transcripts could exceed 1/100 of the number of β-actin transcripts. Although this latter assertion requires a general validation, it has been confirmed by RT-qPCR analyses performed on a limited number of test genes [35–37]. This normalized expression ratio for ubiquitin C was estimated to 0.28 ±0.08% (n = 10) [36], which could explain why the corresponding probes ─ used as positive control in nucleated cells ─ only stained a subpopulation of retPLTs. For IL-1β and BCL3, when given (a minority of the PLT samples in the three studies), the ratios were far below this 1/100 threshold; moreover, in one paper, these genes did not figure on the list of those expressed at a level exceeding 1/10,000 of that of β-actin [36]. Although accurate experimental confirmation could be needed, the absence of IL-1β or BCL3 from the list of genes expressed at a level of above 1/10,000 of that of β-actin [36] is difficult to reconcile with the claims that all PLTs can synthesize IL-1β when incubated with LPS [41,42,46], or that PLT activation results in Bcl3 synthesis, which subsequently allows clot retraction [43]. Further studies will be necessary to clarify what experimental or theoretical methodological bias could explain these conflicting observations and interpretations.

Our *ex vivo* labeling experiments demonstrated that retPLTs display a constitutive active translation system, which nevertheless mostly vanishes within a few hours. In agreement with our data, previous studies have shown that amino acid incorporation was more important in young PLTs [27]. Notably, the electrophoretic profiles of proteins revealed by autoradiography or Coomassie staining indicated that the highest metabolic incorporations corresponded to the most abundant proteins. This suggests that the most biologically active mRNAs encode the major proteins in MKs, and hence their translation in PLTs may not be expected to have a significant biological function. It cannot be excluded that very active proteins could be constitutively biosynthesized after PLT generation, but these molecules remain to be identified.

In clinical practice, the retPLT count is a useful marker of thrombocytopenia, as it indicates an increased production of PLTs in response to their pathological consumption and excludes a defect in thrombopoiesis. Two main techniques based on fluorescent dyes targeting RNA molecules identify the so-called “immature PLT fraction” (IPF) and retPLTs, which are partially correlated, indicating that the two methods investigate different features of young PLTs. In the healthy population, the mean percentages of IPF and reticulated PLTs are 3.4% and 1.4–2.2%, respectively [48]. The IPF can be moderately expanded in coronary artery disease [49], renal transplant recipients [50] or thrombocytopenic neonates [51], or can dramatically rise to 20% or more in PLT consuming diseases such as disseminated intravascular coagulation or autoimmune thrombocytopenia [52]. Higher IPFs have been reported, but could be artifactual, due to interference from apoptotic blood cell fragments [53]. Our work raises the question as to whether the transient constitutive translation in IPF could have biological implications in pathological states accompanied by a dramatically increased number of these PLTs.

Several studies have indicated that retPLTs display increased reactivity in classical functional *in vitro* tests, although investigating the biology of retPLTs *in vivo* is a difficult task [54]. Our experimental system now provides a unique opportunity to investigate, not only *in vitro* but also *in vivo*, the functional characteristics of retPLTs and the biological relevance of translation in PLTs in specific processes, such as the recruitment of young PLTs, or the PLT lifespan. This latter aspect is interesting with regard to PLT transfusion. Indeed, while control PLT counts significantly decreased 24 h after transfusion, a 24–48 h lag time was observed when retPLTs were transfused (Fig 3D). These observations are reminiscent of a clinical assay consisting of the transfusion of PLT preparations with an increased IPF in children undergoing autologous peripheral hematopoietic progenitor cell transplantation, who subsequently experienced reduced transfusion needs [55]. Thus, our experimental system provides a model to assess the *in vivo* behavior of retPLTs and to investigate their potential advantages in transfusion.

## Methods

### Animals

Experiments were performed in 8 to 10 week-old mice carrying an inducible diphtheria toxin receptor gene (CBy.B6-Gt(ROSA)26Sortm1(HBEGF)Awai/J) (iDTR mice), control wild type mice (BALB/CByJ), or in mice carrying the Gt(ROSA)26Sortm4(ACTB-tdTomato,-EGFP)Luo/J allele (Jax® mice 7576), all purchased from Charles River Laboratories (L’Arbresle, France). HR35 mouse strain that ubiquitously express EGFP under beta-actin promoter was kindly provided by P Chambon, Illkirch. PF4-Cre (C57BL/6) mice [14] were kindly provided by R. Skoda. F1 iDTR x PF4-Cre female mice were used in all experiments. To better control the PLT rebounds, experiments were preferentially performed in 2–3 month-old mice. Thrombocytopenia was induced by daily i.p. injection of 100 ng DT (Santa Cruz) for 4 days and checked 3 days later (on day 7). All mice were housed in the animal facilities of the EFS-Alsace (agreement N° D67-482-10). Mice were anesthetized by inhalation of isoflurane before DT administration, small blood sample collection, or PLT transfusion. Animals were sacrificed by inhalation of CO2 (conscious animals) or cervical dislocation (end of experimental procedures on anesthetized animals).

### Ethic statement

The human blood samples were obtained from voluntary and unpaid donors, recruited by EFS-Alsace blood donation center (Etablissement Français du Sang-Alsace; http://www.efs-alsace.fr/), where the research was performed. On each blood collection occasion, the donors signed an informed consent indicating that the samples could be used for research purposes.

Ethical approval for the animal experiments was in accordance with the European Union directive 2010/63/EU. The study was approved by the Regional Ethic Committee for Animal Experimentation of Strasbourg, C.R.E.M.E.A.S. (CEEA 35).

### Mouse PLT preparation and erythrocyte and leukocyte depletion

PLTs were isolated 4 days after the last DT injection (on day 8). Mice were anesthetized with an i.p. injection of ketamine (100 mg/kg) and xylazine (20 mg/kg) and blood was collected from the aorta into acid citrate dextrose anticoagulant. The entire preparation of washed PLTs was performed at room temperature. Mouse blood was centrifuged at 2300g to obtain PLT rich plasma. The PLT count was determined using a Scil Vet abc plus hematology analyzer to adjust the subsequent washes to a concentration of 600,000 PLTs/μL. After incubation for 10 min, PLT rich plasma was centrifuged at 2200g and the PLT pellet was resuspended in Tyrode’s albumin buffer (5 mM Hepes pH 7.35, 0.35% human serum albumin) supplemented with 0.5 μM PGI_2_ and 10 U/mL heparin. After incubation for 10 min, 0.5 μL/mL PGI_2_ (10^−3^ M) was added and the PLTs were centrifuged at 1900g. This washing step was performed a second time and the PLTs were finally resuspended at 300,000/μL in Tyrode’s albumin buffer containing 0.02 U/mL apyrase. These PLT preparations were used for immunofluorescence and *in vitro* translation experiments.

For RNA analyses, blood samples were first centrifuged on Histopaque 1077 (Sigma-Aldrich) supplemented with 0.5 μM PGI_2_ (10^−3^ M) and 10 U/mL heparin at 250g for 30 min, after which washed PLTs were prepared from the interface containing PLTs. Residual numbers of erythrocytes and leukocytes were counted on a Scil Vet abc plus analyzer. The preparations were then depleted of erythrocytes and leukocytes by incubation with Dynabeads coated with the monoclonal antibodies Ter119 and 30-F-11 (anti-mouse CD45) (both from Biolegend; 1 μg antibody/25 μL beads, as recommended), using a ratio of 3 antibody-coated beads per target cell and two serial depletion steps. Depletion was controlled by FC analysis of 10^6^ PLTs (S5 Fig) and the ratio of residual leukocytes to PLTs was always less than 2/10^6^.

For transfusion experiments, washed reticulated PLTs were prepared from three DT-treated mice having PLT counts between 200 and 400 10^3^/μL. The washed PLTs were resuspended at 1.2 10^6^/μL and aliquots of the suspension were injected retro-orbitally into mice expressing tdTomato or EGFP protein (150 μL/mouse). Blood samples were collected 15 min after transfusion (time 0) and then 1, 3, 6, 9, 24, 48 and 72 h later.

In all *ex vivo* incubations, washed PLTs (in Tyrode’s albumin buffer) were finally resuspended at 300,000/μL in Tyrode’s albumin buffer mixed with DMEM (1/1, v/v) supplemented with 0.02 U/mL apyrase.

### Metabolic labeling

Leukocyte- and erythrocyte-depleted washed PLTs were incubated in complete DMEM/Tyrode’s albumin medium (1/1 v/v) at 37°C for various periods of time before sampling (after 10 min, 5 h 30 and 23 h 30). PLT samples were washed using two serial centrifugations at 1900g in cysteine-free DMEM/Tyrode’s albumin buffer supplemented with 0.5 μM PGI_2_, incubated in methionine- and cysteine-free DMEM/Tyrode’s albumin buffer for 30 min at 37°C, washed again using two serial centrifugations in cysteine-free DMEM/Tyrode’s albumin buffer supplemented with 0.5 μM PGI_2_ and then labeled in methionine- and cysteine-free DMEM/Tyrode’s albumin medium supplemented with ^35^S methionine and cysteine (0.5 mCi/10^9^ PLTs/1 mL, Perkin-Elmer) for 30 min at 37°C After labeling, the PLTs were washed using two serial centrifugations in methionine- and cysteine-free DMEM supplemented with 0.5 μM PGI_2_ and platelet pellet was lysed in 100 μL of 2% SDS at 95°C for 5 min. Protein concentrations were determined using a BCA assay (Thermo Scientific) and 25 μg samples were loaded onto SDS polyacrylamide gels (4–15%, Biorad). After electrophoresis, the gels were stained with Coomassie blue G250, fixed, dried and autoradiographed (Amersham Hyperfilm MP, D Dutscher).

### RNA extraction

Blood was collected mice and leukocyte- and erythrocyte-depleted washed PLTs were prepared as described above. About 10^9^ PLTs from untreated animals, or 2 10^8^ from DT-treated mice, were used for each RNA extraction. The cells were centrifuged and lysed in 1 mL of Trizol (Invitrogen, Saint Aubin, France) and RNA was extracted according to the recommended methods. Additional adsorption step on silica matrix (RNeasy, Qiagen) was performed when indicated using manufacturer’s recommended conditions, eluted RNA samples were ethanol precipitated and then dissolved in 5 μl RNase-free water. The RNA concentration was determined on a NanoDrop 2000 spectrophotometer and correlated with the PLT count.

For RNA stability assays, 5 times fewer PLTs were used. Leukocyte- and erythrocyte—depleted PLTs were counted, suspended in a mixture of Tyrode’s albumin buffer and DMEM culture medium (1/1, 300 000 PLTs/μL) and incubated at 37°C for the indicated times. The PLTs were then centrifuged and 100 μL of Trizol reagent was added. Purified MS2 RNA (550 ng/10^8^ reticulated PLTs, Roche Applied Sciences) and 1 μg glycogen (Roche Applied Sciences) were added in the extraction reagent. The extracted RNA was dissolved in water (1 μL/6.8 10^6^ PLTs) and analyzed on a Bioanalyzer 2100 using an RNA 6000 Nano Kit (Agilent). For each sample, the following parameters were derived: the total amount of RNA, proportional to the surface under the RNA curves (S_RNA_), and the time corrected surface under the MS2 peak (S_MS2_). Since a Bioanalyzer analysis of MS2 RNA indicated that 67% of the RNA species lay under the MS2 peak, the surface under the curve which corresponded to purified PLT RNA was S_RNA_- S_MS2_/0.67. To compensate for the experimental variability of the RNA extractions and to compare the samples at each time point (t), we calculated the ratio R(t) = [S_RNA_- S_MS2_/0.67]/S_MS2_. The percentage of remaining RNA in PLTs at each time point was determined using the ratio R(t)/R0.

For experiments with megakaryocyte RNA, lineage-negative bone marrow cells were differentiated in megakaryocytes as previously described [56]. On day 3 of the culture, 80% of the cells were MKs and, RNA was extracted using Trizol reagent and quantified on a Nanodrop spectrophotometer.

## Reverse Transcription and Quantitative PCR

At each time point of the RNA stability assay (time 0, 2, 4, 6 and 24 h, 4 independent RNA from 7 10^6^ platelet (containing control MS2 RNA, 5.50 ng/10^6^ PLTs, see § RNA extraction) experiments), was reverse transcribed using 4 μL iScript^TM^ Reverse Transcription supermix according to the manufacturer instructions (20 μL final volume, Bio-Rad). Quantitative PCR assays were run in SsoAdvanced™ universal SYBR® Green supermix (Bio-Rad) on a Bio-Rad CFX96 (95°C 30s, followed by 40 cycles 95°C 5s, 58°C 30s). The couples of oligonucleotides for MS2, 28S and beta actin were GCAATGCAAGGTCTCCTAAA / AGAAAGATCGCGAGGAAGAT, CCGAAGTTTCCCTCAGGATA / CCAAGACCTCTAATCATTCGC and GAGAAGATCTGGCACCACACC / GCTGGGGTGTTGAAGGTCTC, respectively. Data were analyzed using CFX Manager 3.1 software (Bio-Rad). For each couple of oligonucleotides, quadruplicated calibration curves were first generated from amplification curves of 1/50, 1/500, 1/5000 and 1/50 000 diluted samples. Efficiencies of PCR were deduced and used for relative quantifications. Representative amplification curves obtained with 1/50 diluted samples are displayed on Fig 5B. Triplicated amplification curves obtained with 1/50 diluted samples were used to deduce the relative quantities of the 28S rRNA, beta actin mRNA, and MS2 RNA (ΔCq) and then to calculate the relative normalized expression (ΔΔCq) using MS2 RNA as reference gene and time 0 as control sample. The relative normalized expression of beta actin to 28S rRNA in different samples at time 0 were independently calculated; of note, this normalized expression was independent of the dilution (1/50, 1/500, 1/5000), indicating absence of strong bias (data not shown).

## Human PLT Preparations

Leukocyte-reduced PLT concentrates were prepared from buffy coats containing Citrate Phosphate Dextrose anticoagulant, pooled from five donors, according to the good manufacturing procedures of blood transfusion (TACSI PL kit, Terumo), but without pathogen neutralization step. These PLT concentrates (10^9^ PLTs/mL) were provided by EFS-Alsace in transfusion bags containing a mixture of approximately 70% plasma and 30% Intersol® additive. The absolute number of residual leukocytes, determined by FC [55], was always fewer than 1 leukocyte/10^6^ PLTs. PLT bags were incubated under slow agitation at 22°C or 37°C and at the indicated time points, 50 mL samples were removed from the bags, The pH and gases in the supernatant were measured immediately using an OMNI S analyzer (Roche), while aliquots of the cells were stained with annexin V or anti-P-selectin antibodies and analyzed by flow cytometry. Samples containing 40 10^9^ PLTs were centrifuged at 2200g for 16 min and the pellets were resuspended in 5 mL of Trizol and immediately frozen at -80°C.

### Flow cytometry

PLT samples were incubated for 15 min at room temperature with 1 μM TO (Sigma-Aldrich) as previously described [57] and 1 μg/mL Alexa 647-conjugated anti-GP1bβ antibody (RAM1). The samples were then diluted in 20 volumes of 1% paraformaldehyde and analyzed by FC (LSRFortessa™ cell analyzer, BD Biosciences) within 45 min. The PLT gate was defined using the strategies described Fig 3C for transfused PLTs, or alternatively S3B Fig for other conditions. TO^bright^ and TO^dim^ gates were defined on resting PLTs from control mice (S3B Fig). Representative dot plots of TO/SSC staining are shown (S3A and S3B Fig). The fate of transfused retPLTs in mice expressing tdTomato was determined after blood sampling and TO and RAM1-A647 staining.

### Immunostaining and analyses

PLT samples were fixed in 3% paraformaldehyde for 15 min (30 000 PLTs/μL). Fixed PLTs were allowed to sediment on poly L-lysin coated coverslips for 15 min, permeabilized in 0.2% BSA and 0.05% saponin containing buffer, and processed for intracellular immunofluorescence staining as usual. The anti-rRNA mAb Y10b (5 μg/mL, mouse IgG3, Novus Biologicals) was used to label ribosomes and revealed with A647-conjugated goat anti-mouse IgG. After a mouse serum blocking step, the plasma membranes of PLTs were stained with an A488-conjugated anti-GP1bβ antibody. Immunolabeling was examined under a Leica SP5-AOBS confocal microscope.

To facilitate visualization of the internal staining of PLTs, wide-field image of cells stained with the anti-GPIbβ mAb RAM1 were processed to define the positions occupied by the PLTs (Fig 4A). Images of anti-GP1bβ and anti-rRNA labeled cells were analyzed using Image J software to calculate the mean fluorescence intensity of the anti-rRNA labeling (per unit area) in individual PLTs. For each condition, at least 500 PLTs were analyzed.

*In situ* detection of transcripts was performed using RNAscope 2.5 kit (ACDbio) and HD-assay RED detection reagent. PLT rich plasma were prepared from saline- and DT-receiving animals and checked by FC after TO and RAM1 labeling. Sample preparation and labeling with transcript-specific probes were performed according manufacturer recommended procedures, except for the boiling step and the counterstaining with hematoxylin that were omitted. Briefly, PLTs were fixed for 60 min at 37°C in 10% Neutral Buffered Formalin. Fixed PLTs were then washed in PBMC-Wash (ACDbio), subsequently suspended in 70% ethanol (30 000 PLTs/μL) and cytospun on SuperfrostTM Plus glass slides (Thermofisher) coated with Cell-Tak (Corning). Then, cells were treated for 30 min at 40°C with the protease reagent, before staining with mouse beta actin- or ubiquitin C-specific probes, or the negative control probe (dapB, *Bacillus subtilis*). Hybridized probes were revealed using alkaline phosphatase and HD-assay RED substrate, then slides were washed in water and PBS. Immunofluorescence was performed as usual with 10 μg/mL Alexa 488-conjugated RAM1 mAb, leukocyte nuclei were stained with DAPI (140 ng/ml), samples were mounted in ProLong™ antifade reagent, then bright field and fluorescent images were acquired with a Leica DMI4000 microscope. Images were acquired with a Photometrics CCD camera (Cool-SNAP HQ Monochrome).Micrographs were processed with Image J, the numbers of RAM1+ cells, positively stained or not with RNA probes, were manually counted. In the case of the analysis of actin transcripts, cells strongly labeled with the actin mRNA probes but poorly labeled with RAM1 mAb, likely corresponding to PLTs based on the bright field micrographs and the absence of associated DAPI fluorescence, were also counted. For each condition, between four to six series of images were analyzed.

### Transmission electron microscopy

PLT and bone marrow samples were fixed for 60 min in 0.1 M sodium cacodylate buffer containing 2.5% glutaraldehyde. The samples were then rinsed and post-fixed for 1 h at 4°C with 1% osmium tetroxide in the same buffer. After additional washing in sodium cacodylate buffer, the samples were dehydrated in increasing concentrations of ethanol and embedded in Epon. The resin was polymerized at 50°C for 2 days. Ultrathin sections (100 nm), stained with lead citrate and uranyl acetate, were examined under a CM 120 BioTwin electron microscope (120 kV).

Megakaryocytes were counted on whole transversal sections of TEM preparations and the mean numbers of cells per section and per square of the grid were calculated, giving an estimation of the mean density of MKs per unit area (corresponding to one 14,400 μm2 square of the grid) [58]. MKs at stages I, II, III and IV were identified according to their distinctive ultrastructural features. Stage I corresponded to 10 to 15 μm diameter cells with a large nucleus, stage II to 15 to 30 μm diameter cells containing PLT-specific granules, stage III to mature MKs displaying a well-developed demarcation membrane system with well-defined PLT territories and a peripheral zone, and stage IV to naked nuclei.

### Statistical analyses

Data were analyzed using Graphpad Prism 5 software. One or two way ANOVA followed by a Bonferroni post-hoc test, or t-tests, were used, depending on the number of conditions.

## Supporting Information

S1 Fig Increased MK density after recovery. PF4-cre-iDTR mice received four daily administration of saline (-DT, upper row) or DT (+DT, lower row). Four days after the last dose, bone marrow were flushed from femurs, fixed and analyzed by TEM as described in the methods. Stars indicate MK nuclei. Scale bar, 20 μm.
(PDF)

S2 Fig Ultrastructure of TO^bright^ platelets. Washed platelets from saline or DT-treated F1 iDTR x PF4-Cre mice were fixed and processed for Epon embedding and microscopy analysis as described in the main section of the article. Representative views of cells from saline- (-DT) and DT-treated (+DT) animals are shown. ER, endoplasmic reticulum; G, Golgi apparatus; N, nuclear remnants. Scale bar: 1μm.
(PDF)

S3 Fig Specificity of TO and Y10b labeling. (A) Washed PTLs (200 000/μl) from DT-treated mice were stained as described in the main section (i.e. incubated with TO and A647-conjugated RAM1 mAb, then fixed and analyzed by flow cytometry, Standard staining). Alternatively, PLTs were fixed in paraformaldehyde and permeabilized in PBS containing 0.05% saponin, 0.2% BSA, before staining with TO and A647-conjugated RAM1 mAb, and then, analyzed by flow cytometry (Fixed and permeabilized); alternatively, the fixed and permeabilized PLTs were incubated for 30min at room temperature in absence (-RNase) or presence (+RNase) of RNase A (100 μg/ml), stained with TO and A647-conjugated RAM1 mAb and then analyzed by flow cytometry. PLT gate was defined using FCS and RAM1 parameters (not shown), then TO^dim^ and TO^bright^ subsets were defined using two combinations of gates adjusted for the analysis of control PLTs, one for PLTs stained under standard conditions (left dot plots), the other for fixed and permeabilized PLTs (other dot plots. Representative dot plots of TO/SSC staining are shown, the percentage of TO^bright^ platelets and the MFI of TO^dim^ and TO^bright^ PLTs are indicated (n = 3). (B) To check the impact of PLT degranulation on TO staining, A555-conjugated RAM1, A647-conjugated anti P-selectin mAbs (0.5 and 1 μg/ml, respectively) and 1U/ml of thrombin were added to washed platelets (30 000/μl). PLTs were incubated 10 min at 37°C, after which recombinant hirudin was added (10U/ml). PLTs were then labeled with TO and analyzed by FC using the standard protocol. Two combinations of gates, adjusted for the analysis of resting or activated control PTLs were designed (right dot plots). Percentages of TO^dim^ and TO^bright^ subsets among all PLTs and, their respective MFIs, are indicated. Activated PLTs were analyzed using the two different types of gates. Histograms represent the MFI and the percentages of resting (R) or activated (A) TO^dim^ and TO^bright^ PLTs from saline- or DT-treated animals (white and grey bars, respectively). Homogeneous and dotted bars correspond to analyses using the gates adjusted for resting or activated PLTs, respectively. Results are the mean ± SEM in three independent experiments. (C) On day 8, washed platelets from DT-treated mice were fixed, permeabilized and stained with the mAb Y10b as described in Fig 4. Alternatively, the permeabilized platelets were treated at room temperature for 30 min with RNase (100 μg/mL) in permeabilization buffer, before staining with Y10b as described in Fig 4. Micrographs of immunostained platelets were processed as described in Fig 4 and identical parameters were used for image acquisition and processing. Scale bar: 10 μm.
(PDF)

S4 Fig Phenotype of platelets incubated *in vitro*. (A) Washed platelets from saline- or DT-treated mice were incubated *in vitro* at 22°C or 37°C for the indicated times (see Fig 3A). Activated GPIIbIIIa expression was determined by flow cytometry and the percentages of positive cells are shown. (B) Washed PLTs from DT- treated animals were fixed and processed for Epon embedding and microscopy analysis as described in the main section of the article. Two preparations were analyzed, freshly isolated PTLs or PLTs incubated at 37°C for 24h in Tyrode albumin buffer/DMEM (50/50) medium. Damaged platelets are denoted with asterisks.
(PDF)

S5 Fig Control of leukocyte and erythrocyte depletion. Washed platelets were depleted of erythrocytes and leukocytes as described in the methods. At each step, the platelet preparations were stained with an Alexa-488-conjugated anti-Gp1bβ mAb (RAM1), Alexa-647-conjugated control IgG (table, C) and anti-erythrocyte (Ter119) or anti-leukocyte (30-F-11) mAbs and analyzed by FC. Gates corresponding to platelets (P), erythrocytes (E) and leukocytes (L), as defined before depletion (upper row), were applied to the other staining combinations after depletion (lower rows). For the samples obtained after two depletion steps, at least 10^6^ events in the platelet gate, defined on the FSC-A/ Gp1bβ dot plot, were acquired before analysis. The numbers of events in the P, E and L gates are indicated and the figure shows the analysis of a representative erythrocyte- and leukocyte-depleted platelet preparation.
(PDF)

S6 Fig Quality of RNA extracted from *in vitro*-differentiated MKs and control or retPLTs. RNAs from *in vitro*-differentiated MKs or from leukocyte- and erythrocyte-depleted PLTs were Trizol extracted and further purified on silica matrix and then quality-checked using a Bioanalyzer 2100 and an RNA 6000 Nano kit (Agilent). On day 8 of the protocol, as described Fig 1A, PLTs from saline- and DT-treated mice were analyzed. (A) Representative profiles of RNA from *in vitro* differentiated MKs and PLTs from DT- or saline-treated animals. (B) Distribution of RIN and 28S/18S RNA values from retPLTs. (C) Percentage of retPLTs vs 28S/18S RNA ratio plot. (D) Percentage of retPLT vs platelet RNA content (fg/platelet). The y values are represented on a two-scale axis, a log2 scale up to 1 fg/platelet and a linear scale from 5 to 45 fg/platelet. For DT-treated animals, each RNA sample (n = 7) corresponds to one mouse, while for untreated animals each sample (n = 7) was extracted from a platelet pool from 4–6 mice. Before RNA extraction, platelet counts were recorded using a Scil Vet abc plus hematology analyzer and the percentage of TO^bright^ platelets was determined by FC.
(PDF)

## References

[1] Jackson SP, Nesbitt WS, Westein E (2009) Dynamics of platelet thrombus formation. J Thromb Haemost 7 Suppl 1: 17–20. 10.1111/j.1538-7836.2009.03401.x 19630759

[2] Herter JM, Rossaint J, Zarbock A (2014) Platelets in inflammation and immunity. J Thromb Haemost 12: 1764–1775. 10.1111/jth.12730 25224706

[3] Rowley JW, Schwertz H, Weyrich AS (2012) Platelet mRNA: the meaning behind the message. Curr Opin Hematol 19: 385–391. 10.1097/MOH.0b013e328357010e 22814651 PMC3670814

[4] Weyrich AS, Schwertz H, Kraiss LW, Zimmerman GA (2009) Protein synthesis by platelets: historical and new perspectives. J Thromb Haemost 7: 241–246. 10.1111/j.1538-7836.2008.03211.x 18983498 PMC3027201

[5] Pillitteri D, Bassus S, Boller K, Mahnel R, Scholz T, Westrup D, et al (2007) Thrombin-induced interleukin 1beta synthesis in platelet suspensions: impact of contaminating leukocytes. Platelets 18: 119–127. 17365860 10.1080/09537100600800792

[6] Gnatenko DV, Cupit LD, Huang EC, Dhundale A, Perrotta PL, Bahou WF (2005) Platelets express steroidogenic 17beta-hydroxysteroid dehydrogenases. Distinct profiles predict the essential thrombocythemic phenotype. Thromb Haemost 94: 412–421. 16113833 10.1160/TH05-01-0037

[7] Ingram M, Coopersmith A (1969) Reticulated platelets following acute blood loss. Br J Haematol 17: 225–229. 5806433 10.1111/j.1365-2141.1969.tb01366.x

[8] Robinson M, MacHin S, Mackie I, Harrison P (2000) In vivo biotinylation studies: specificity of labelling of reticulated platelets by thiazole orange and mepacrine. Br J Haematol 108: 859–864. 10792296 10.1046/j.1365-2141.2000.01939.x

[9] Dale GL, Friese P, Hynes LA, Burstein SA (1995) Demonstration that thiazole-orange-positive platelets in the dog are less than 24 hours old. Blood 85: 1822–1825. 7535589

[10] Buttarello M, Plebani M (2008) Automated blood cell counts: state of the art. Am J Clin Pathol 130: 104–116. 10.1309/EK3C7CTDKNVPXVTN 18550479

[11] Meintker L, Haimerl M, Ringwald J, Krause SW (2013) Measurement of immature platelets with Abbott CD-Sapphire and Sysmex XE-5000 in haematology and oncology patients. Clin Chem Lab Med 51: 2125–2131. 10.1515/cclm-2013-0252 23800658

[12] Kieffer N, Guichard J, Farcet JP, Vainchenker W, Breton-Gorius J (1987) Biosynthesis of major platelet proteins in human blood platelets. Eur J Biochem 164: 189–195. 3830180 10.1111/j.1432-1033.1987.tb11010.x

[13] Buch T, Heppner FL, Tertilt C, Heinen TJ, Kremer M, Wunderlich FT, et al (2005) A Cre-inducible diphtheria toxin receptor mediates cell lineage ablation after toxin administration. Nat Methods 2: 419–426. 15908920 10.1038/nmeth762

[14] Tiedt R, Schomber T, Hao-Shen H, Skoda RC (2007) Pf4-Cre transgenic mice allow the generation of lineage-restricted gene knockouts for studying megakaryocyte and platelet function in vivo. Blood 109: 1503–1506. 17032923 10.1182/blood-2006-04-020362

[15] Berger G, Hartwell DW, Wagner DD (1998) P-Selectin and platelet clearance. Blood 92: 4446–4452. 9834252

[16] Zucker-Franklin D (2003) Atlas of Blood Cells, Function and Pathology. Milano: edi.ermes.

[17] Bruns I, Lucas D, Pinho S, Ahmed J, Lambert MP, Kunisaki Y, et al (2014) Megakaryocytes regulate hematopoietic stem cell quiescence through CXCL4 secretion. Nat Med 20: 1315–1320. 10.1038/nm.3707 25326802 PMC4258871

[18] Zhao M, Perry JM, Marshall H, Venkatraman A, Qian P, He XC, et al (2014) Megakaryocytes maintain homeostatic quiescence and promote post-injury regeneration of hematopoietic stem cells. Nat Med 20: 1321–1326. 10.1038/nm.3706 25326798

[19] Robinson MS, Mackie IJ, Khair K, Liesner R, Goodall AH, Savidge GF, et al (1998) Flow cytometric analysis of reticulated platelets: evidence for a large proportion of non-specific labelling of dense granules by fluorescent dyes. Br J Haematol 100: 351–357. 9488626 10.1046/j.1365-2141.1998.00563.x

[20] Manning KL, McDonald TP (1997) C3H mice have larger spleens, lower platelet counts, and shorter platelet lifespans than C57BL mice: an animal model for the study of hypersplenism. Exp Hematol 25: 1019–1024. 9293898

[21] Schroeder A, Mueller O, Stocker S, Salowsky R, Leiber M, Gassmann M, et al (2006) The RIN: an RNA integrity number for assigning integrity values to RNA measurements. BMC Mol Biol 7: 3 16448564 10.1186/1471-2199-7-3 PMC1413964

[22] Ault KA, Rinder HM, Mitchell J, Carmody MB, Vary CP, Hillman RS (1992) The significance of platelets with increased RNA content (reticulated platelets). A measure of the rate of thrombopoiesis. Am J Clin Pathol 98: 637–646. 1281383 10.1093/ajcp/98.6.637

[23] Garden GA, Hartlage-Rubsamen M, Rubel EW, Bothwell MA (1995) Protein masking of a ribosomal RNA epitope is an early event in afferent deprivation-induced neuronal death. Mol Cell Neurosci 6: 293–310. 7496633 10.1006/mcne.1995.1023

[24] Lerner EA, Lerner MR, Janeway CA Jr., Steitz JA (1981) Monoclonal antibodies to nucleic acid-containing cellular constituents: probes for molecular biology and autoimmune disease. Proc Natl Acad Sci U S A 78: 2737–2741. 6789322 10.1073/pnas.78.5.2737 PMC319432

[25] Thomas MG, Martinez Tosar LJ, Loschi M, Pasquini JM, Correale J, Kindler S, et al (2005) Staufen recruitment into stress granules does not affect early mRNA transport in oligodendrocytes. Mol Biol Cell 16: 405–420. 15525674 10.1091/mbc.E04-06-0516 PMC539183

[26] Abou Elela S, Nazar RN (1997) Role of the 5.8S rRNA in ribosome translocation. Nucleic Acids Res 25: 1788–1794. 9108162 10.1093/nar/25.9.1788 PMC146658

[27] Booyse FM, Zschocke D, Hoveke TP, Rafelson ME Jr. (1971) Studies on human platelets. IV. Protein synthesis in maturing human platelets. Thromb Diath Haemorrh 26: 167–176. 5165248

[28] Bruce IJ, Kerry R (1987) The effect of chloramphenicol and cycloheximide on platelet aggregation and protein synthesis. Biochem Pharmacol 36: 1769–1773. 3579972 10.1016/0006-2952(87)90236-x

[29] Wadhawan V, Karim ZA, Mukhopadhyay S, Gupta R, Dikshit M, Dash D (2004) Platelet storage under in vitro condition is associated with calcium-dependent apoptosis-like lesions and novel reorganization in platelet cytoskeleton. Arch Biochem Biophys 422: 183–190. 14759606 10.1016/j.abb.2003.12.024

[30] Mason KD, Carpinelli MR, Fletcher JI, Collinge JE, Hilton AA, Ellis S, et al (2007) Programmed anuclear cell death delimits platelet life span. Cell 128: 1173–1186. 17382885 10.1016/j.cell.2007.01.037

[31] Nikolov EN, Dineva BB, Dabeva MD, Nikolov TK (1987) Turnover of ribosomal proteins in regenerating rat liver after partial hepatectomy. Int J Biochem 19: 159–163. 3569644 10.1016/0020-711x(87)90326-0

[32] Nikolov EN, Dabeva MD, Nikolov TK (1983) Turnover of ribosomes in regenerating rat liver. Int J Biochem 15: 1255–1260. 6628827 10.1016/0020-711x(83)90215-x

[33] Defoiche J, Zhang Y, Lagneaux L, Pettengell R, Hegedus A, Willems L, et al (2009) Measurement of ribosomal RNA turnover in vivo by use of deuterium-labeled glucose. Clin Chem 55: 1824–1833. 10.1373/clinchem.2008.119446 19696118

[34] Wada Y, Li D, Merley A, Zukauskas A, Aird WC, Dvorak HF, et al (2011) A multi-gene transcriptional profiling approach to the discovery of cell signature markers. Cytotechnology 63: 25–33. 10.1007/s10616-010-9315-8 20972619 PMC3021151

[35] Rowley JW, Oler AJ, Tolley ND, Hunter BN, Low EN, Nix DA, et al (2011) Genome-wide RNA-seq analysis of human and mouse platelet transcriptomes. Blood 118: e101–111. 10.1182/blood-2011-03-339705 21596849 PMC3193274

[36] Londin ER, Hatzimichael E, Loher P, Edelstein L, Shaw C, Delgrosso K, et al (2014) The human platelet: strong transcriptome correlations among individuals associate weakly with the platelet proteome. Biol Direct 9: 3 10.1186/1745-6150-9-3 24524654 PMC3937023

[37] Bray PF, McKenzie SE, Edelstein LC, Nagalla S, Delgrosso K, Ertel A, et al (2013) The complex transcriptional landscape of the anucleate human platelet. BMC Genomics 14: 1 10.1186/1471-2164-14-1 23323973 PMC3722126

[38] Ingolia NT, Lareau LF, Weissman JS (2011) Ribosome profiling of mouse embryonic stem cells reveals the complexity and dynamics of mammalian proteomes. Cell 147: 789–802. 10.1016/j.cell.2011.10.002 22056041 PMC3225288

[39] Hase K, Fujiwara Y, Kikuchi H, Aizawa S, Hakuno F, Takahashi S, et al (2015) RNautophagy/DNautophagy possesses selectivity for RNA/DNA substrates. Nucleic Acids Res 43: 6439–6449. 10.1093/nar/gkv579 26038313 PMC4513860

[40] Fujiwara Y, Furuta A, Kikuchi H, Aizawa S, Hatanaka Y, Konya C, et al (2013) Discovery of a novel type of autophagy targeting RNA. Autophagy 9: 403–409. 10.4161/auto.23002 23291500 PMC3590259

[41] Lindemann S, Tolley ND, Dixon DA, McIntyre TM, Prescott SM, Zimmerman GA, et al (2001) Activated platelets mediate inflammatory signaling by regulated interleukin 1beta synthesis. J Cell Biol 154: 485–490. 11489912 10.1083/jcb.200105058 PMC2196422

[42] Shashkin PN, Brown GT, Ghosh A, Marathe GK, McIntyre TM (2008) Lipopolysaccharide is a direct agonist for platelet RNA splicing. J Immunol 181: 3495–3502. 18714022 10.4049/jimmunol.181.5.3495 PMC2551315

[43] Weyrich AS, Denis MM, Schwertz H, Tolley ND, Foulks J, Spencer E, et al (2007) mTOR-dependent synthesis of Bcl-3 controls the retraction of fibrin clots by activated human platelets. Blood 109: 1975–1983. 17110454 10.1182/blood-2006-08-042192 PMC1801071

[44] Rosenwald IB, Pechet L, Han A, Lu L, Pihan G, Woda B, et al (2001) Expression of translation initiation factors elF-4E and elF-2alpha and a potential physiologic role of continuous protein synthesis in human platelets. Thromb Haemost 85: 142–151. 11204566

[45] Lindemann S, Tolley ND, Eyre JR, Kraiss LW, Mahoney TM, Weyrich AS (2001) Integrins regulate the intracellular distribution of eukaryotic initiation factor 4E in platelets. A checkpoint for translational control. J Biol Chem 276: 33947–33951. 11431478 10.1074/jbc.M104281200

[46] Denis MM, Tolley ND, Bunting M, Schwertz H, Jiang H, Lindemann S, et al (2005) Escaping the nuclear confines: signal-dependent pre-mRNA splicing in anucleate platelets. Cell 122: 379–391. 16096058 10.1016/j.cell.2005.06.015 PMC4401993

[47] Bray PF, McKenzie SE, Edelstein LC, Nagalla S, Delgrosso K, Ertel A, et al (2013) The complex transcriptional landscape of the anucleate human platelet. BMC Genomics 14: 1 10.1186/1471-2164-14-1 23323973 PMC3722126

[48] Hoffmann JJ (2014) Reticulated platelets: analytical aspects and clinical utility. Clin Chem Lab Med 52: 1107–1117. 10.1515/cclm-2014-0165 24807169

[49] Cesari F, Marcucci R, Caporale R, Paniccia R, Romano E, Gensini GF, et al (2008) Relationship between high platelet turnover and platelet function in high-risk patients with coronary artery disease on dual antiplatelet therapy. Thromb Haemost 99: 930–935. 10.1160/TH08-01-0002 18449424

[50] Cesari F, Marcucci R, Gori AM, Caporale R, Fanelli A, Paniccia R, et al (2010) High platelet turnover and reactivity in renal transplant recipients patients. Thromb Haemost 104: 804–810. 10.1160/TH10-02-0124 20694276

[51] Cremer M, Weimann A, Schmalisch G, Hammer H, Buhrer C, Dame C (2010) Immature platelet values indicate impaired megakaryopoietic activity in neonatal early-onset thrombocytopenia. Thromb Haemost 103: 1016–1021. 10.1160/TH09-03-0148 20216981

[52] Kickler TS, Oguni S, Borowitz MJ (2006) A clinical evaluation of high fluorescent platelet fraction percentage in thrombocytopenia. Am J Clin Pathol 125: 282–287. 16393688 10.1309/50H8-JYHN-9JWC-KAM7

[53] Briggs C, Longair I, Kumar P, Singh D, Machin SJ (2012) Performance evaluation of the Sysmex haematology XN modular system. J Clin Pathol 65: 1024–1030. 10.1136/jclinpath-2012-200930 22851510

[54] Dusse LM, Freitas LG (2015) Clinical applicability of reticulated platelets. Clin Chim Acta 439: 143–147. 10.1016/j.cca.2014.10.024 25451948

[55] Parco S, Vascotto F (2012) Application of reticulated platelets to transfusion management during autologous stem cell transplantation. Onco Targets Ther 5: 1–5. 10.2147/OTT.S27883 22334789 PMC3278260

[56] Strassel C, Eckly A, Leon C, Moog S, Cazenave JP, Gachet C, et al (2012) Hirudin and heparin enable efficient megakaryocyte differentiation of mouse bone marrow progenitors. Exp Cell Res 318: 25–32. 10.1016/j.yexcr.2011.10.003 22008103

[57] Matic GB, Chapman ES, Zaiss M, Rothe G, Schmitz G (1998) Whole blood analysis of reticulated platelets: improvements of detection and assay stability. Cytometry 34: 229–234. 9822309 10.1002/(sici)1097-0320(19981015)34:5<229::aid-cyto4>3.0.co;2-2

[58] Eckly A, Strassel C, Cazenave JP, Lanza F, Leon C, Gachet C (2012) Characterization of megakaryocyte development in the native bone marrow environment. Methods Mol Biol 788: 175–192. 10.1007/978-1-61779-307-3_13 22130708

