# Supplementary figures and images for: Time-Dependent Decay of mRNA and Ribosomal RNA during Platelet Aging and Its Correlation with Translation Activity
Source: PLoS One. 2016 Jan 25;11(1):e0148064. doi: 10.1371/journal.pone.0148064 (PMC4726520; doi:10.1371/journal.pone.0148064)

S1 Fig. Increased MK density after recovery.

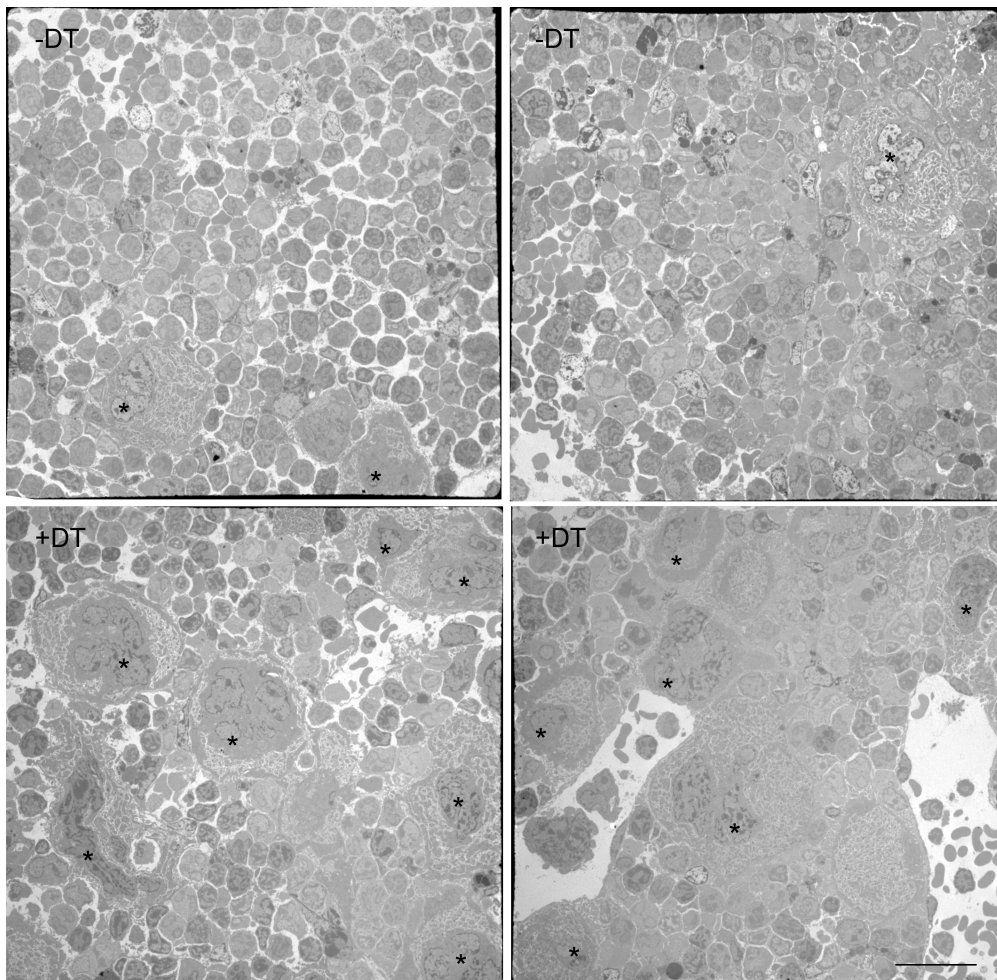

Supplement: S1 Fig — PF4-cre-iDTR mice received four daily administration of saline (-DT, upper row) or DT (+DT, lower row). Four days after the last dose, bone marrow were flushed from femurs, fixed and analyzed by TEM as described in the methods. Stars indicate MK nuclei. Scale bar, 20 μm. (PDF) [file pone.0148064.s001.pdf]

S2 Fig. Ultrastructure of TObright platelets.

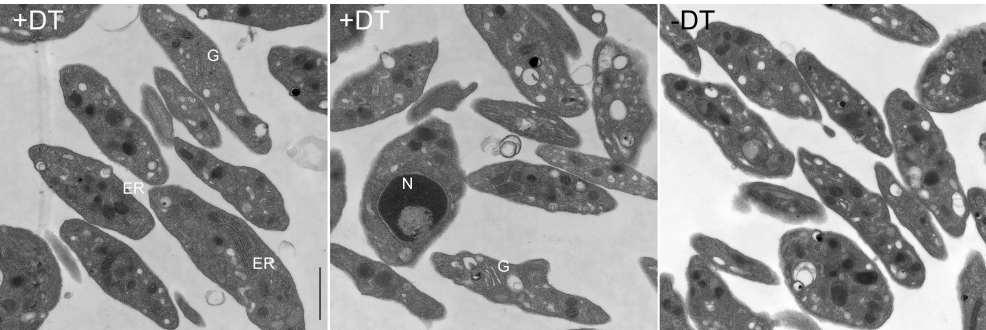

Supplement: S2 Fig — Washed platelets from saline or DT-treated F1 iDTR x PF4-Cre mice were fixed and processed for Epon embedding and microscopy analysis as described in the main section of the article. Representative views of cells from saline- (-DT) and DT-treated (+DT) animals are shown. ER, endoplasmic reticulum; G, Golgi apparatus; N, nuclear remnants. Scale bar: 1μm. (PDF) [file pone.0148064.s002.pdf]

# S4 Fig. Phenotype of platelets incubated in vitro

A

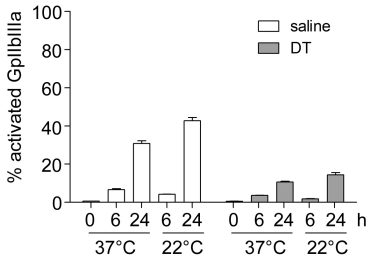

B

0 h

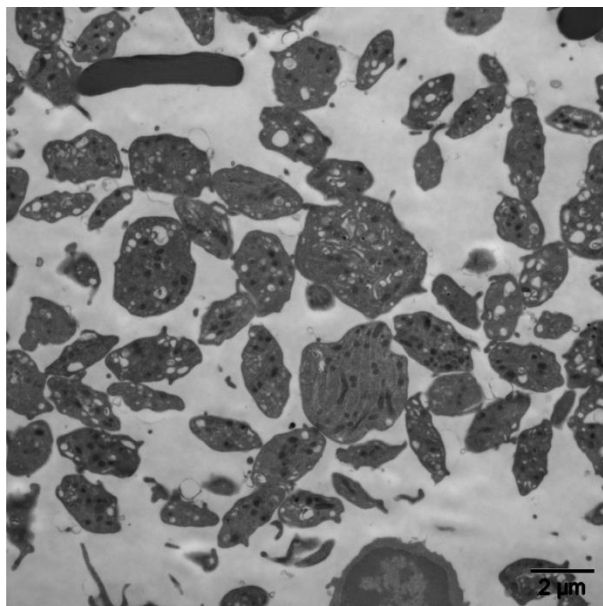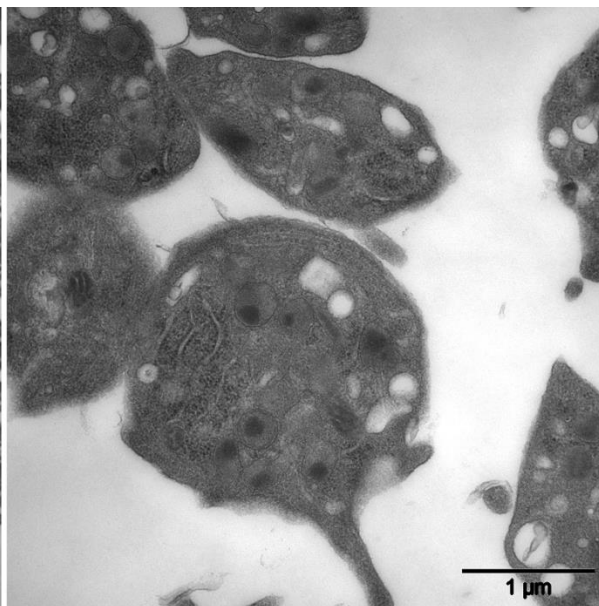

24 h

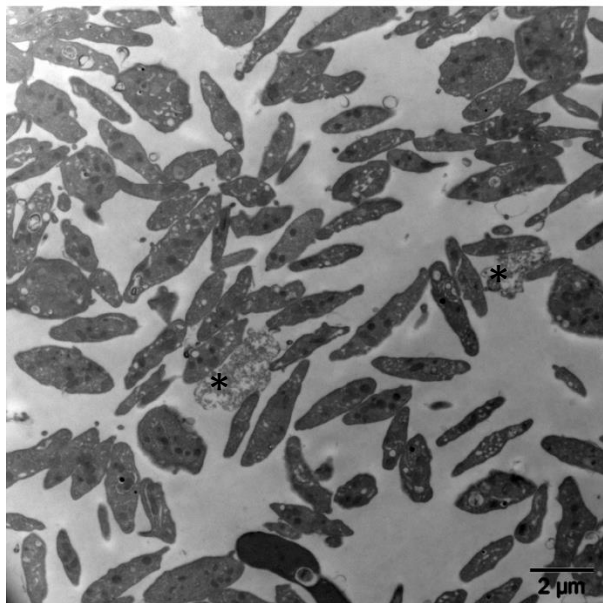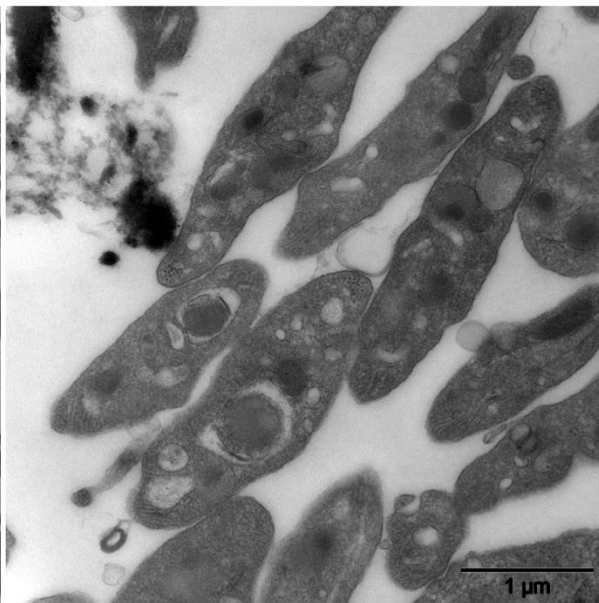

Supplement: S4 Fig — (A) Washed platelets from saline- or DT-treated mice were incubated in vitro at 22°C or 37°C for the indicated times (see Fig 3A). Activated GPIIbIIIa expression was determined by flow cytometry and the percentages of positive cells are shown. (B) Washed PLTs from DT- treated animals were fixed and processed for Epon embedding and microscopy analysis as described in the main section of the article. Two preparations were analyzed, freshly isolated PTLs or PLTs incubated at 37°C for 24h in Tyrode albumin buffer/DMEM (50/50) medium. Damaged platelets are denoted with asterisks. (PDF) [file pone.0148064.s004.pdf]

**S5 Fig. Control of leukocyte and erythrocyte depletion.**

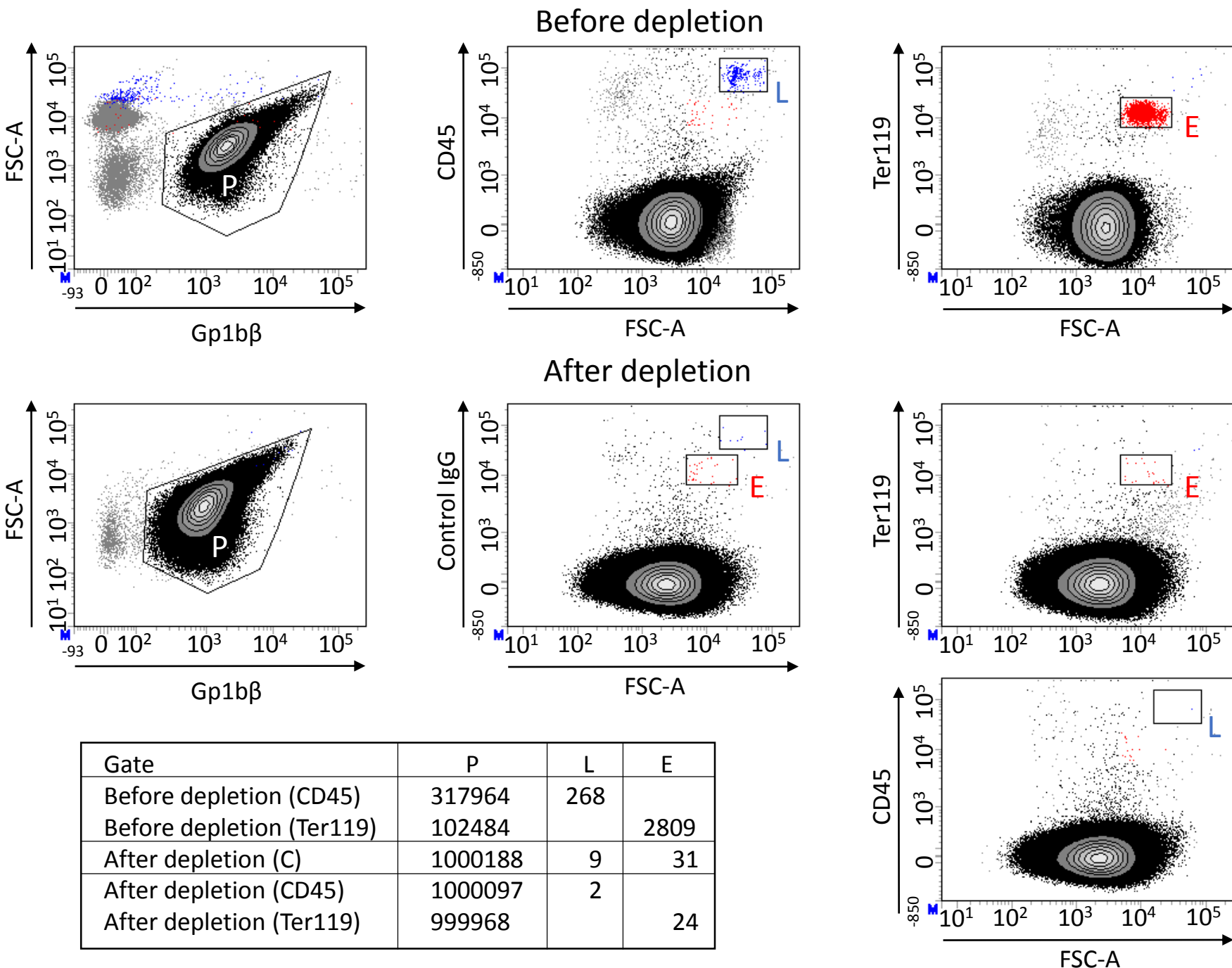

Supplement: S5 Fig — Washed platelets were depleted of erythrocytes and leukocytes as described in the methods. At each step, the platelet preparations were stained with an Alexa-488-conjugated anti-Gp1bβ mAb (RAM1), Alexa-647-conjugated control IgG (table, C) and anti-erythrocyte (Ter119) or anti-leukocyte (30-F-11) mAbs and analyzed by FC. Gates corresponding to platelets (P), erythrocytes (E) and leukocytes (L), as defined before depletion (upper row), were applied to the other staining combinations after depletion (lower rows). For the samples obtained after two depletion steps, at least 106 events in the platelet gate, defined on the FSC-A/ Gp1bβ dot plot, were acquired before analysis. The numbers of events in the P, E and L gates are indicated and the figure shows the analysis of a representative erythrocyte- and leukocyte-depleted platelet preparation. (PDF) [file pone.0148064.s005.pdf]

S6 Fig. Quality of RNA extracted from in vitro-differentiated MKs and control or retPLTs.

A

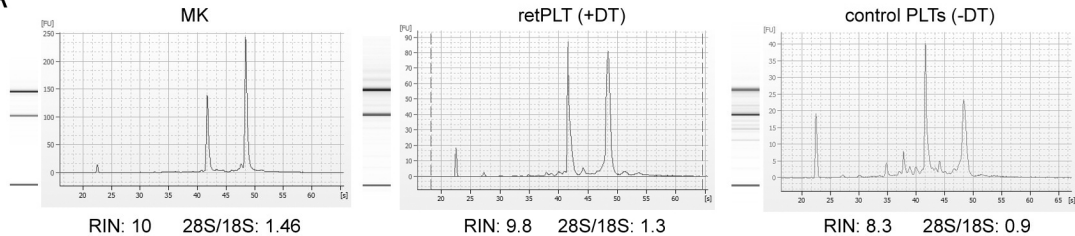

B

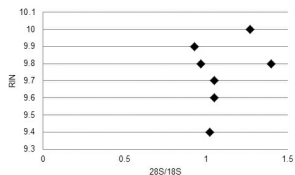

C

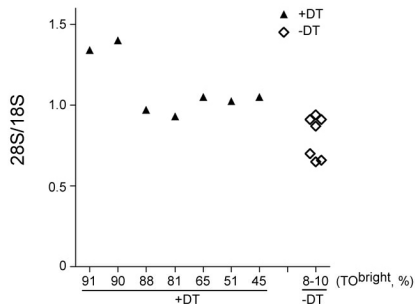

D

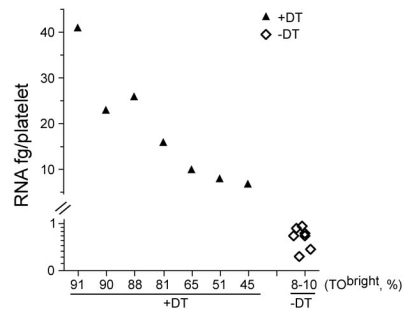

Supplement: S6 Fig — RNAs from in vitro-differentiated MKs or from leukocyte- and erythrocyte-depleted PLTs were Trizol extracted and further purified on silica matrix and then quality-checked using a Bioanalyzer 2100 and an RNA 6000 Nano kit (Agilent). On day 8 of the protocol, as described Fig 1A, PLTs from saline- and DT-treated mice were analyzed. (A) Representative profiles of RNA from in vitro differentiated MKs and PLTs from DT- or saline-treated animals. (B) Distribution of RIN and 28S/18S RNA values from retPLTs. (C) Percentage of retPLTs vs 28S/18S RNA ratio plot. (D) Percentage of retPLT vs platelet RNA content (fg/platelet). The y values are represented on a two-scale axis, a log2 scale up to 1 fg/platelet and a linear scale from 5 to 45 fg/platelet. For DT-treated animals, each RNA sample (n = 7) corresponds to one mouse, while for untreated animals each sample (n = 7) was extracted from a platelet pool from 4–6 mice. Before RNA extraction, platelet counts were recorded using a Scil Vet abc plus hematology analyzer and the percentage of TObright platelets was determined by FC. (PDF) [file pone.0148064.s006.pdf]
