# Supplementary material for: Time-Dependent Decay of mRNA and Ribosomal RNA during Platelet Aging and Its Correlation with Translation Activity
Source: PLoS One. 2016 Jan 25;11(1):e0148064. doi: 10.1371/journal.pone.0148064 (PMC4726520; doi:10.1371/journal.pone.0148064)

S3 Fig. Specificity of TO and Y10b labeling.

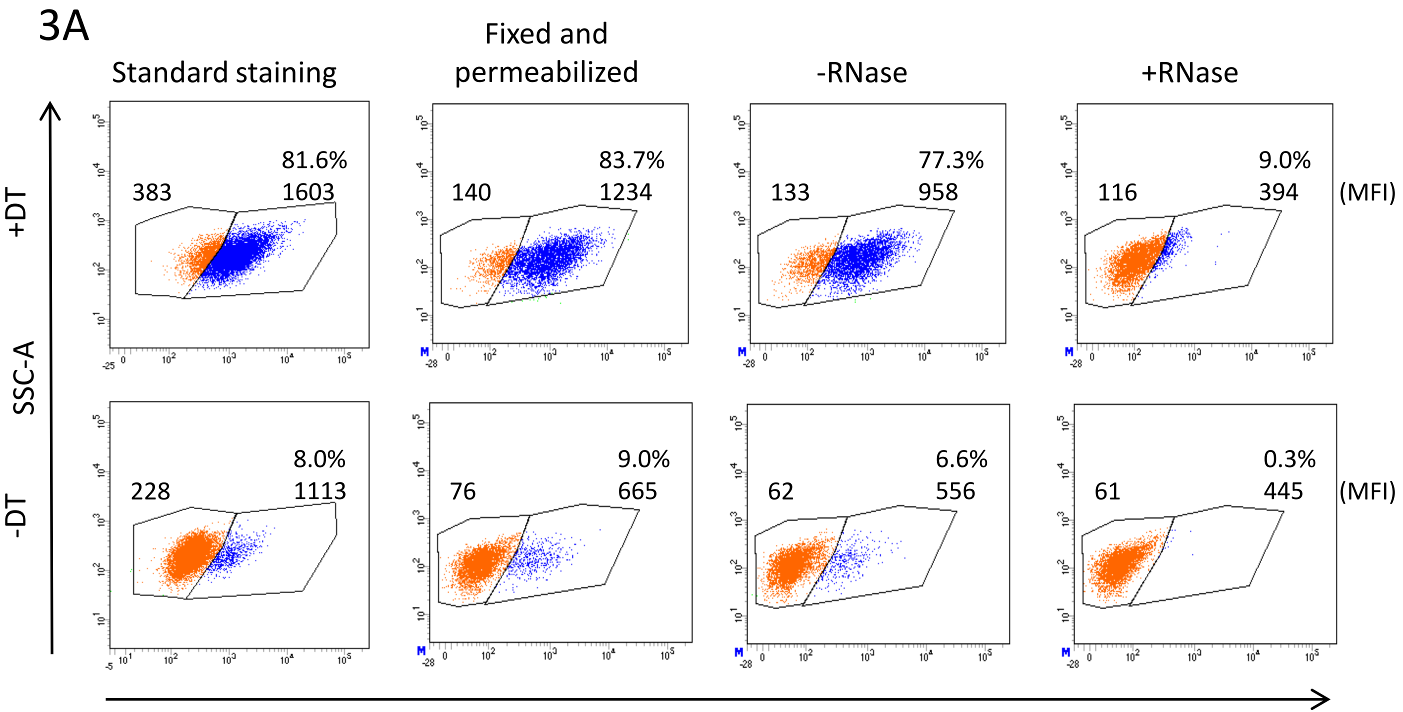

3B

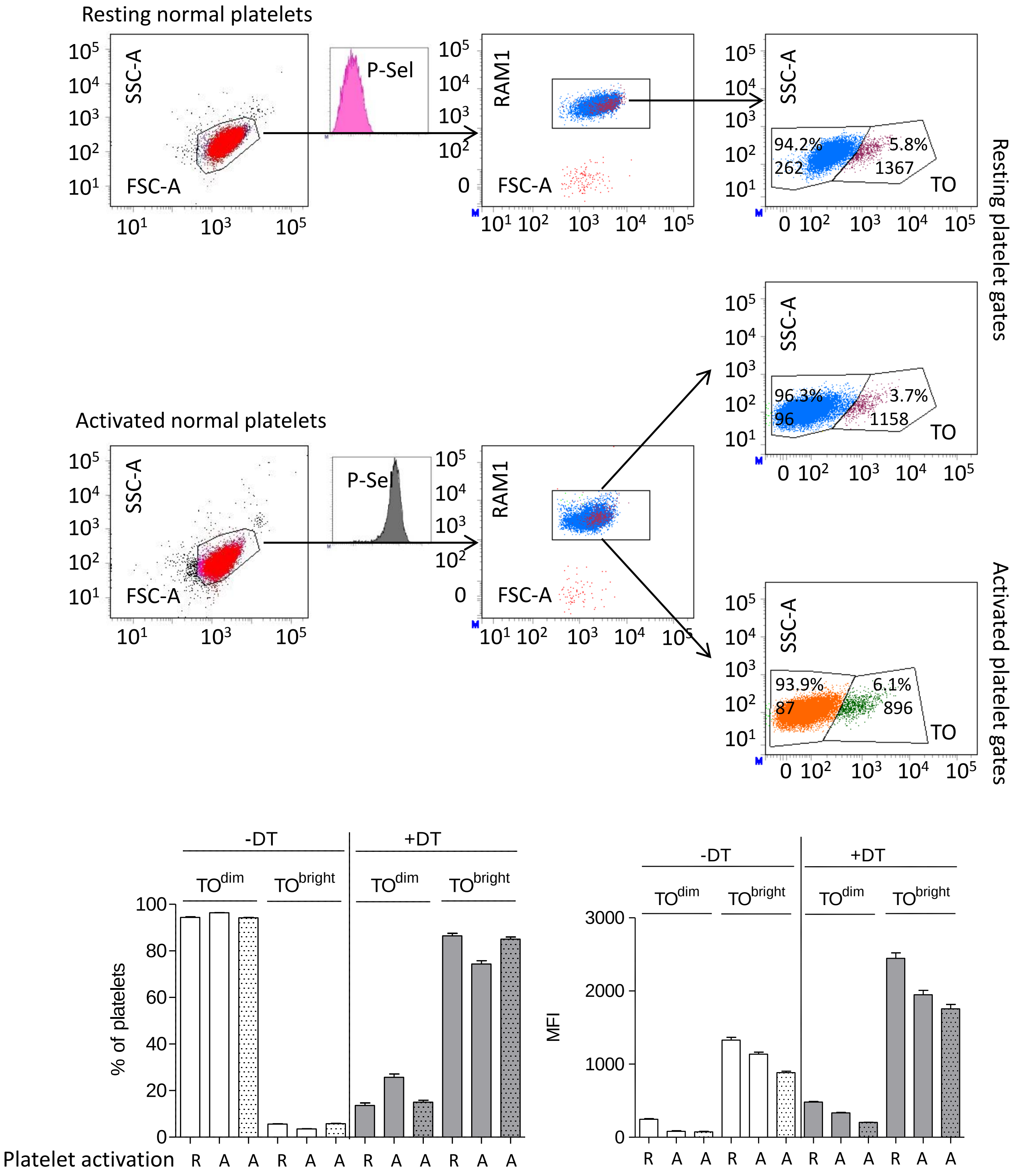
Resting normal platelets
SSC-A
FSC-A
P-Sel
RAM1
FSC-A
SSC-A
94.2%
262
5.8%
1367
TO
Resting platelet gates
SSC-A
96.3%
96
3.7%
1158
TO
Activated normal platelets
SSC-A
FSC-A
P-Sel
RAM1
FSC-A
SSC-A
93.9%
87
6.1%
896
TO
Activated platelet gates
-DT
+DT
TOdim
TObright
% of platelets
MFI
Platelet activation
R A A R A A R A A R A A

3C

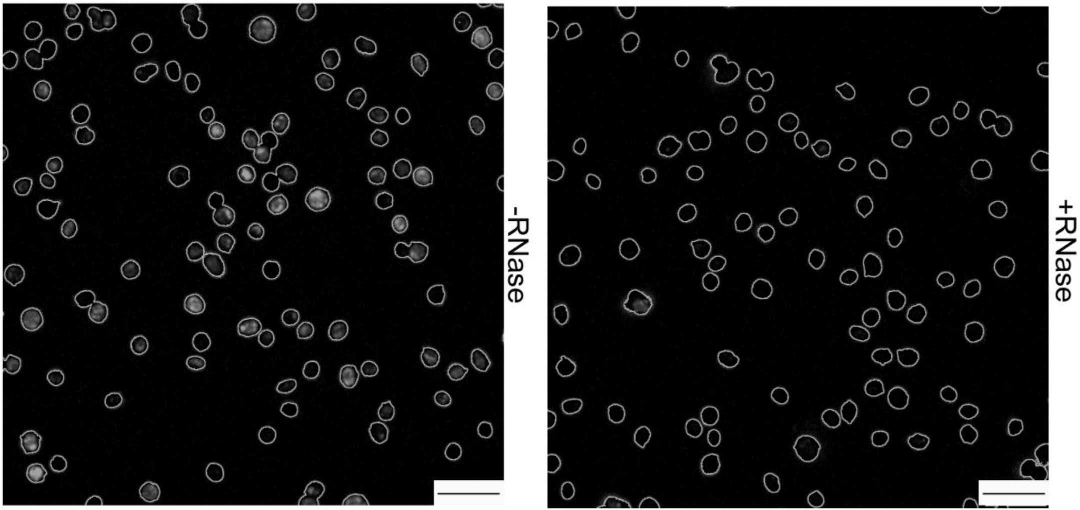

Supplement: S3 Fig — (A) Washed PTLs (200 000/μl) from DT-treated mice were stained as described in the main section (i.e. incubated with TO and A647-conjugated RAM1 mAb, then fixed and analyzed by flow cytometry, Standard staining). Alternatively, PLTs were fixed in paraformaldehyde and permeabilized in PBS containing 0.05% saponin, 0.2% BSA, before staining with TO and A647-conjugated RAM1 mAb, and then, analyzed by flow cytometry (Fixed and permeabilized); alternatively, the fixed and permeabilized PLTs were incubated for 30min at room temperature in absence (-RNase) or presence (+RNase) of RNase A (100 μg/ml), stained with TO and A647-conjugated RAM1 mAb and then analyzed by flow cytometry. PLT gate was defined using FCS and RAM1 parameters (not shown), then TOdim and TObright subsets were defined using two combinations of gates adjusted for the analysis of control PLTs, one for PLTs stained under standard conditions (left dot plots), the other for fixed and permeabilized PLTs (other dot plots. Representative dot plots of TO/SSC staining are shown, the percentage of TObright platelets and the MFI of TOdim and TObright PLTs are indicated (n = 3). (B) To check the impact of PLT degranulation on TO staining, A555-conjugated RAM1, A647-conjugated anti P-selectin mAbs (0.5 and 1 μg/ml, respectively) and 1U/ml of thrombin were added to washed platelets (30 000/μl). PLTs were incubated 10 min at 37°C, after which recombinant hirudin was added (10U/ml). PLTs were then labeled with TO and analyzed by FC using the standard protocol. Two combinations of gates, adjusted for the analysis of resting or activated control PTLs were designed (right dot plots). Percentages of TOdim and TObright subsets among all PLTs and, their respective MFIs, are indicated. Activated PLTs were analyzed using the two different types of gates. Histograms represent the MFI and the percentages of resting (R) or activated (A) TOdim and TObright PLTs from saline- or DT-treated animals (white and grey bars, respectively). [file pone.0148064.s003.pdf]
